# Multivariate tools to investigate the spatial contaminant distribution in a highly anthropized area (Gulf of Naples, Italy)

**DOI:** 10.1007/s11356-022-19989-z

**Published:** 2022-04-09

**Authors:** Matilda Mali, Antonella Di Leo, Santina Giandomenico, Lucia Spada, Nicola Cardellicchio, Maria Calò, Alessandra Fedele, Luciana Ferraro, Alfonsa Milia, Monia Renzi, Francesca Massara, Tommaso Granata, Letizia Moruzzi, Francesco Paolo Buonocunto

**Affiliations:** 1 DICATECh, Politecnico Di Bari, via Orabona, 4 I-70125 Bari, Italy; 2 Water Research Institute, National Research Council, Taranto, Italy; 3 Institute of Marine Sciences, National Research Council, Naples, Italy; 4 Department of Life Science, L. Giorgieri, 10, 34127 Trieste, Italy; 5 CONISMA - Consorzio Nazionale Interuniversitario Per Le Scienze del Mare, Roma , Italy; 6 Terna S.P.A. - Rete Elettrica Nazionale Roma - Viale Egidio Galbani, 70, Roma, Italy; 7 CESI S.P.a. - Centro Elettronico Sperimentale Italiano, Milano, Italy

**Keywords:** Hazard degree, Complex dataset, Marine sediments, Contamination, Multivariate analyses, Gulf of Naples

## Abstract

The Gulf of Naples located in a high anthropized coastal area is subjected to an infrastructural intervention for the installation of a submarine power pipeline. In order to evaluate the distribution of contaminants in the seafloor sediments, a preliminary study has been conducted in the area using multivariate techniques. The statistic approach was performed to gain insights on the occurrence of organic and inorganic contaminants within the area, aiming to identify the relevant hot spots. Three geographical sub-areas influenced by different contaminant association were recognized: Torre Annunziata (TA), Capri (CA), and middle offshore (MO). TA and CA resulted marked by a severe contamination pattern due to anthropogenic pressures. In addition, the influence of the depositional basin in governing the contamination trend has been pointed out. The supervised technique PLS_DA resulted to be a powerful tool in addressing the complexity of the huge dataset acquired during the marine survey, highlighting the main trends in the variability of quality indicators, orienting thus the deeper investigations during follow-up monitoring activities.

**Supplementary Information:**

The online version contains supplementary material available at 10.1007/s11356-022-19989-z.

## Introduction

Environmental monitoring of highly anthropized areas demands for a deep analysis of the different environmental compartments (water, bottom sediments, and biota) and the determination of numerous bio-geo-chemical parameters, because of the huge impact of organic and inorganic substances constantly released into these environments.

Sediments are often used as the best indicator for assessing the quality of marine environment as they are integrated components of the aquatic ecosystems and recognized as sinks of all contaminants accumulated in the bottom seafloor generated by the different sources: atmospheric pollution or direct discharges of contaminants in the sea-water column. Additionally, sediments also act as a source of contaminants by natural and anthropogenic processes (Chapman 1986, 2007; DelValls et al. 1998; Wenning et al. 2005; Chapman 2007; Piva et al. 2011; Rubio et al. 2011; Chapman and Smith 2012; Regoli et al. 2013; Chapman et al. 2013; Mali et al. 2016, 2017a, b, c, 2018; 2019; Cotecchia et al. 2021).

Different indices have been developed for contamination assessment tools for the monitoring of sediments in aquatic ecosystems (MacDonald et al. 1996; Long et al. 1995a,b, 2006; Casado-Martìnez et al. 2009; Varol 2011; Chapman and Smith 2012; Chapman et al. 2013; Mali et al. 2016; 2017a,b). Nevertheless, when the investigation campaign addresses multi-source contamination such as the case of highly anthropized areas, the interpretation of the generated large dataset becomes awkward (Cotecchia et al. 2021). Definition of the hot spot areas and the recognition of factors controlling the spatial distribution of contaminants require data analysis approaches able to summarize the information content of the huge dataset and to individuate “summary indices” that can be easily visualized and understand. In this way, a comprehensive evaluation of the severity of sediment-associated contamination, especially for the decision-making purposes, would be easily accessible.

The study area of the present work is located in the Campania region whose coastal area is marked by a high demographic pressure, in particular offshore the Sarno River Plain. Sarno River Plain is one of the most polluted areas in Europe due to widespread industrialization and intensive agriculture activity in the surrounding area. Previous analyses have documented the heavy contamination derived from the Sarno River due to the discharges of human and industrial wastes (Albanese et al. 2010, 2013; Arienzo et al. 2017). Montuori and Triassi (2012) reported that the discharges of polycyclic aromatic hydrocarbons (PAHs) from the Sarno River to the Gulf of Naples reach approximately 8530 g/d. On the other side, the Sorrento Peninsula and Capri are subjected to an increasing tourism demand which is also of high concern.

This marine area, in the framework of an infrastructural project related with the “interconnection of the Campania islands to the national electric power transmission network,” was involved in the installation of a three-pole submarine viaduct, with a length of approximately 30 km, connecting the Capri Island to Torre Annunziata.

In order to characterize the area from an environmental point of view, surficial sediments were selected from 158 sites in the marine-coastal area overlooking the island of Capri, the coast of Torre Annunziata, and the offshore interconnection area between the two landings. Different environmental indicators, according to Italian legislation (D.L. 152/2006), were selected for the contamination assessment: percentage of water (W%), specific weight (Ps), grain size, total nitrogen (TN) and phosphorus (TP), total organic carbon (TOC), and trace elements (As, Cd, Crtot, Cu, Hg, Ni, Pb, Zn) including also Fe and Mn as major elements, the 16 priority EPA congeners of polycyclic aromatic hydrocarbons (PAHs), total petroleum hydrocarbons (TPHs), volatile organic compounds (VOCs), 19 congeners of polychlorinated biphenyls (PCBs), and organotin compounds (OTs) including -mono, -di and -tri butyltin congeners.

The hugeness, complexity, and variability of the dataset obtained make its interpretation very difficult. Statistical techniques, hierarchical cluster analysis (HCA), principal component analyses (PCA), and supervised partial least squares discriminant analysis (PLS_DA) techniques were therefore used for the elaboration of the complex dataset. HCA is a cluster analysis approach which seeks to build a hierarchy of clusters and associated with PCA belongs to the so-called unsupervised pattern recognition methods. Both HCA and PCA can be applied to any dataset without requiring or supposing any preliminary knowledge of the information hidden in the dataset (Massart and Kaufman 1983; Einax et al. 1997). On the other hand, partial least square (PLS), a supervised pattern recognition method, can maximize the amount of the variance explained by the principal components of PCA and thus can support construction of predictive models (Gredilla et al. 2012) finding linear regression model through the projection of the predicted variables and the observable variables on the new PLS Space.

The aim of the present research is the application of multivariate statistical analyses for (i) the identification of hot spot areas with peculiar pollution pattern; (ii) the recognition of the discriminant contaminants in each of the hot spot areas; (iii) the correlation between contamination trends and physico-chemical features of sediments; and (iv) to establish the influence of natural versus anthropogenic factors on the spatial distribution of the contamination.

In the present work, we coupled different multivariate techniques aiming at identifying the differences and similarities between clusters hidden in the generated datasets, extracting principal components (new variance descriptors) able to simplify the systematic variation of original dataset without loss of information (Wang et al. 2009; Pedersen et al. 2015; Mali et al. 2017a).

## Materials and methods

### Geographical, geo-morphologic, and environmental setting

**Fig. 1:**
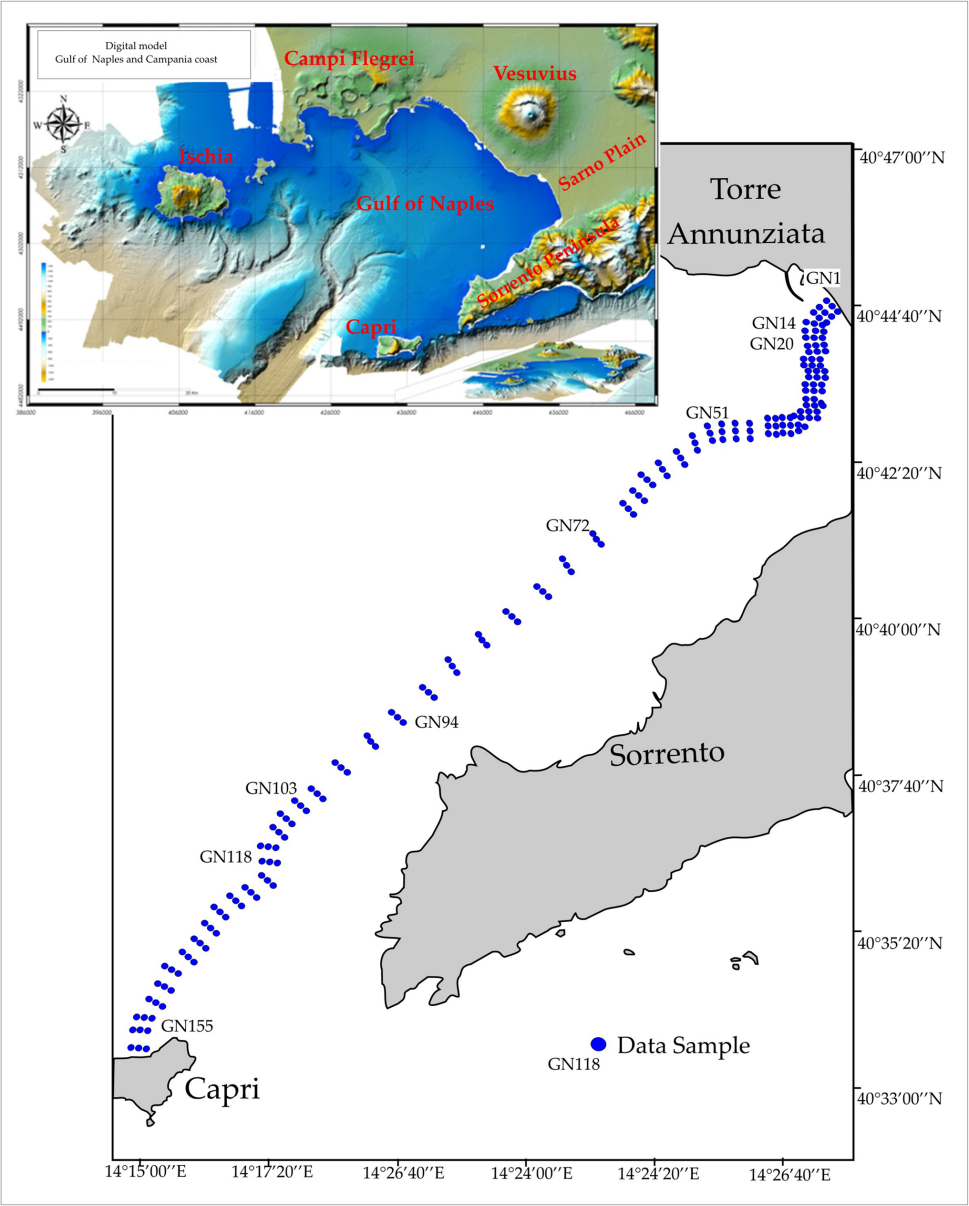
The mapping strategy in the studied area. The location of the 158 sites in blue color, alongside 62 transepts (three/two samples for each transect)

The study area belongs to the south-eastern part of the Gulf of Naples, and it is bounded by the Vesuvius volcano, the Sarno Plain, and Sorrento Peninsula-Capri Island (Fig. 1). The Vesuvius is a 1.2-km-high stratovolcano emplaced over the last 25,000 years (Rosi et al. 1986) along the Campania coast and affected by multiple lateral collapses (Milia et al. 2003). It is one of the most active volcanoes in the world, with more than 80 eruptions listed in a worldwide directory of volcanic activity (Simkin and Siebert 1994). Vesuvius products include basic, silica-undersaturated, potassic rocks, ranging from tephrite to phonolite, and less alkaline basaltic trachyandesites (Ayuso et al. 1998). The Sarno Plain corresponds to a sedimentary basin filled with several meters of volcano-clastic, alluvial, fluvial-marine, and lagoon deposits Middle Pleistocene to Present in age (Milia et al. 2017). The plain is crossed by the Sarno River whose hydrographic basin covers an area of approximately 500 km^2^, which consequently strongly impacts the corresponding discharge area on the Gulf of Naples. Finally, the Sorrento Peninsula-Capri Island is an elongated ENE-WSW relief mainly made up of sedimentary rocks, such as limestone and dolostone Meso-Cenozoic in age, locally covered by Miocene clastic deposits in the south-western part and pyroclastic deposits of the Campanian Ignimbrite (39 ka; De Vivo et al. 2001) in the Sorrento area (Fig. 1).

The Gulf of Naples is characterized by a wide continental shelf extending until the depth of 150–200 m and displays erosional, depositional and volcanic features (Milia 1999). In particular, the southern shelf, close to the Sorrento Peninsula-Capri Island, is affected by erosional surfaces affecting the Meso-Cenozoic carbonate rocks, covered locally by the pyroclastic deposits of the Campanian Ignimbrite. Clastic deposits, corresponding to the deltaic deposits of the Sarno River, are present offshore the Sarno Plain (Sacchi et al. 2005).

The whole Sarno Plain is characterized by a high density of population and is one of the most polluted areas in Europe due to the widespread industrialization and intensive agriculture. The majority of the coastal zone and many inland areas are strongly urbanized and industrialized. In particular, the Sarno Plain is used for intensive agriculture. Furthermore, the area is characterized by the presence of numerous leather tanneries and chemical–pharmaceutical, engineering, and manufacturing firms (Arienzo et al. 2001; De Pippo et al. 2006).

Over the last decade, some studies performed in the onshore area highlighted a serious contamination degree due to metals (e.g., Albanese et al. 2013), organochlorine pesticides (OCPs), polychlorinated biphenyls (PCBs), or polycyclic aromatic hydrocarbons (PAHs) (Qu et al. 2018, 2019).

### Sampling

The sediment samples, for a total of 158 sites, were collected during the month of October of 2014 in the Gulf of Naples (GN), prior to the power submarine pipeline installation. The sampling stations were located in 62 transepts; these are made up of two/three samples spaced 100 m apart. Each transept is sampled with a frequency ranging from 200 m, offshore Torre Annunziata, to 1000 m, in the distal part. The transects are orthogonal to the tract of submarine pipeline that connects the Torre Annunziata shoreline (TA) and Capri (CA) according to the mapping strategy shown in Fig. 1.

Surface sediments samples were collected by Van Veen grab (surface sampling area of 5.84 dm^2^) equipped with upper doors, in order to collect undisturbed sampling at the surface level of 0–2 cm. The sampling scheme, water depth, and geographical coordinates according to the WGS84 reference system are shown in Fig. 1 and Table S1 (Supporting Information) respectively.

After sampling, an aliquot of sediments was stored in clean polyethylene bags and frozen at + 4/ + 6 °C for physical analyses. A second aliquot was stored in decontaminated HDPE (high-density polyethylene) containers at – 18 °C/ − 25 °C for chemical analyses.

### Analytical methods

The samples were subjected to physical analysis to determine the percentage of water (W%), specific weight (Ps) (Table S2), and grain size of particles according to Wentworth classification: coarse (4096–2.00 mm), sand (2.00–0.0625 mm), silt (0.0625–0.0039 mm), and clay (0.0039–0.00006 mm) (Wentworth 1922).

Samples for grain size analysis were treated with H_2_O_2_ solution, aiming at removing organic constituents and support deflocculating, then washed and dried at 40 °C, and analyzed following the ICRAM Manual Procedure (ICRAM 2001). The coarse fraction (> 63 μm) was sieved using ASTM series sieves, while the finest fraction (< 63 μm) was analyzed by means of laser diffraction granulometer (laser particle-size analyzer).

TOC and TN were determined according to ICRAM Method, (ICRAM 2001). Briefly, an aliquot (about 25 mg) of air-dried samples was acidified with HCl 1 M, heated at 60 °C, and processed by the Thermo Electron Flash EA1112 nitrogen and carbon analyzer where He and O_2_ flow were set to 300 and 250 mL/min, respectively. TP content was assessed following APAT IRSA-CNR 4110 (APAT IRSA-CNR 4110 2003) spectrophotometric method using a Varian Cary 50 spectrometer. TOC, TN, and TP were reported in mg/kg dw.

For chemical determinations, sediments were freeze-dried and successively sieved to exclude particles > 2 mm.

Trace elements were determined by inductively coupled plasma mass spectrometry (ICP-MS) (EPA Method 6020B 2014) after strong acid digestion (HF + HNO_3_) of sediments in a microwave oven (EPA Method 3052 2004), aiming at dissolving even the aluminosilicate structures and leaching in the elutriate the fraction of trace elements entrapped in this mineralogic fraction. PAHs, PCBs, and TPHs were extracted with a microwave oven (EPA Method 3546 2007). PAHs and PCBs after specific clean-up (EPA Method 3630C 1996, EPA Method 3620C 2014) were determined by gas chromatography accoupled to mass spectrometry (GC–MS) (EPA Method 8270E 2014), while TPHs (C > 12) were purified and analyzed by gas chromatography with flame ionization detection (GC-FID) according to UNI EN ISO 16703 Method (UNI EN ISO 16703:^2011^), and ISPRA Method 75/2011 (ISPRA 2011). As to PAHs, 16 compounds defined as high-priority pollutants by US EPA were considered; they include naphthalene (Naph), acenaphthene (Ace), acenaphthylene (Acy), phenanthrene (Phe), anthracene (Anthr), fluoranthene (Fluo), Florene (Fl), benz[a] anthracene (BaA), chrysene (Chry), benz[b]fluoranthene (BbF), benz[k]fluoranthene (BkF), benzo[a]pyrene (BaP), benzo(g,h,i)perylene (B.g.h.i.P), dibenz[a,h]anthracene (D.B.a.h.A), fluorene (fl), indeno[1,2,3-cd] (IND), and pyrene (Pyr). For PCBs, the congeners analyzed were those indicated in the Italian Ministerial Decree 173/2016 (D.Lgs. 173/2016): PCB 28, PCB 52, PCB 77, PCB 81, PCB 101, PCB 118, PCB 126, PCB 128, PCB 138, PCB 153, PCB 156, PCB169, PCB 180. Analysis of VOCs (C < 12) was carried out using Headspace GC–MS (EPA Method 5021A 2014, EPA Method 8270D 2014). OTs were determined, after derivatization with the Grignard reagent, in GC–MS (Morabito et al. 1995; ICRAM 2001). A detailed description of these methods with data on QC is provided in the Annex S1 of the Supplementary Information.

### Statistical analyses

SIMCA 16 Program (16, MKS Umetrics AB, Sweden) was used to perform multivariate statistical analyses (Eriksson et al. 2013). The whole dataset is constituted by 158 sites, which geographic coordinates are reported in Table S1, countersigned with GNn code, where “*n*” is the number order of the sampling site. The numbering starts from Torre Annunziata coastline (GN1) up to coastline of Capri (GN158) (Fig. 1). Fifty-seven variables were considered: the absolute values of concentration of As, Cd, Cr_tot_, Cu, Hg, Ni, Pb, Zn, Fe and Mn, PAHs, PCBs and OT compounds and their respective sum (reported as ΣPAHs, ΣPCBs and ΣOTs), TOC, TN, TP, VOCs, and TPHs; the four Wentworth size classes. The percentage of water (W%) content and specific weight (Ps) are also considered (Table S2). For analytes whose concentrations were below the detection limit, half of the detection limit was used as table value. Prior to statistical analysis, variables were also log-transformed and scaled to unit-variance aiming at reducing the influence of the unequal variance of the considered variables (contaminants concentrations, textural data, and nutrient concentrations data) and providing them the same weight in the PCA-relevant axes calculated by the technique (Van Den Berg et al. 2006). Information about other pre-treatment processes and diagnostic tools adopted during multivariate analysis (*R*^2^, *R*^2^*X*, *Q*^2^, DModX, misclassification list) are reported in Eriksson et al. 2013).

## Results and discussion

### Univariate data analysis

#### Granulometry of sediments

**Fig. 2:**
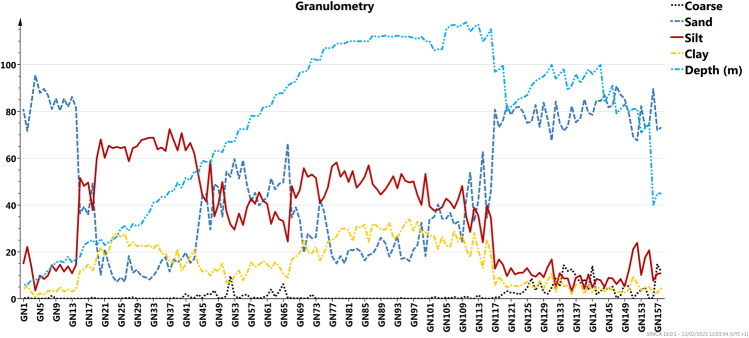
Distribution of granulometric fractions, including water depth, in each site. The granulometric fractions are expressed in W%, while depth in meter

The study area displays different sediment features in terms of grain size (Fig. 2 and Table S2) with a prevalence of sandy and silty fraction ranging in the intervals of 7 ÷ 96%w and 3 ÷ 72%w respectively. The gravel fraction varies in the range of 0–15 W% and clay fraction ranges from 0.7 to 34 W%. Nevertheless, the analysis of the whole investigated area reveals that in some sub-areas different grain sizes coexist with different percentages. Sediments, collected in correspondence of Torre Annunziata (TA) and Capri (CA) shoreline, are mainly composed of sand (up to 96 W% on TA and up to 91 W% on CA) and by a minor amount of silty and clay fraction. The central part of the track displays a heterogeneous granulometric content with a silty fraction that represents the main component for two sectors up to 70 W% between the sites GN20-GN51 and up to 60 W% between the sites GN72 -GN111. The gravel fraction is present along Capri shoreline and between GN52 up to GN71, where the gravel content exceeds 10 W%.

#### Organic matter and nutrients (TOC, TN, TP)

The content of organic matter in sediments is expressed in terms of TOC (Van Nugteren et al. 2009). The distribution of TOC within sediments spans an unusually large range from 0.01 up to 5.88 W%. The highest concentrations of TOC are present offshore Torre Annunziata (TA) shoreline where TOC reached 5.88 W%. This value is higher respect to that detected in an adjacent area, corresponding offshore Naples, ranging between 0.05 and 4% (Sprovieri et al. 2007), whereas in the Adriatic and Ionian marine sediments stays below 1 W% (Bartolini 2015). The concentration of phosphorous (TP) results below the detection limit in almost all stations, except for two coastal tracts: the area between stations GN15 and GN46 off Torre Annunziata (500 ≤ TP ≤ 1534 mg /kg d.s) and the area between sites GN91 and GN106 (500 ≤ TP ≤ 1022 mg/kg d.s) in the middle offshore. The graph of TP trend is reported in Figure S3 of the Supporting information. The pair correlation (TOC vs TP) is to a less extent with respect to the pair TOC vs TN) (*R*^2^ = 0.647).

**Fig. 3:**
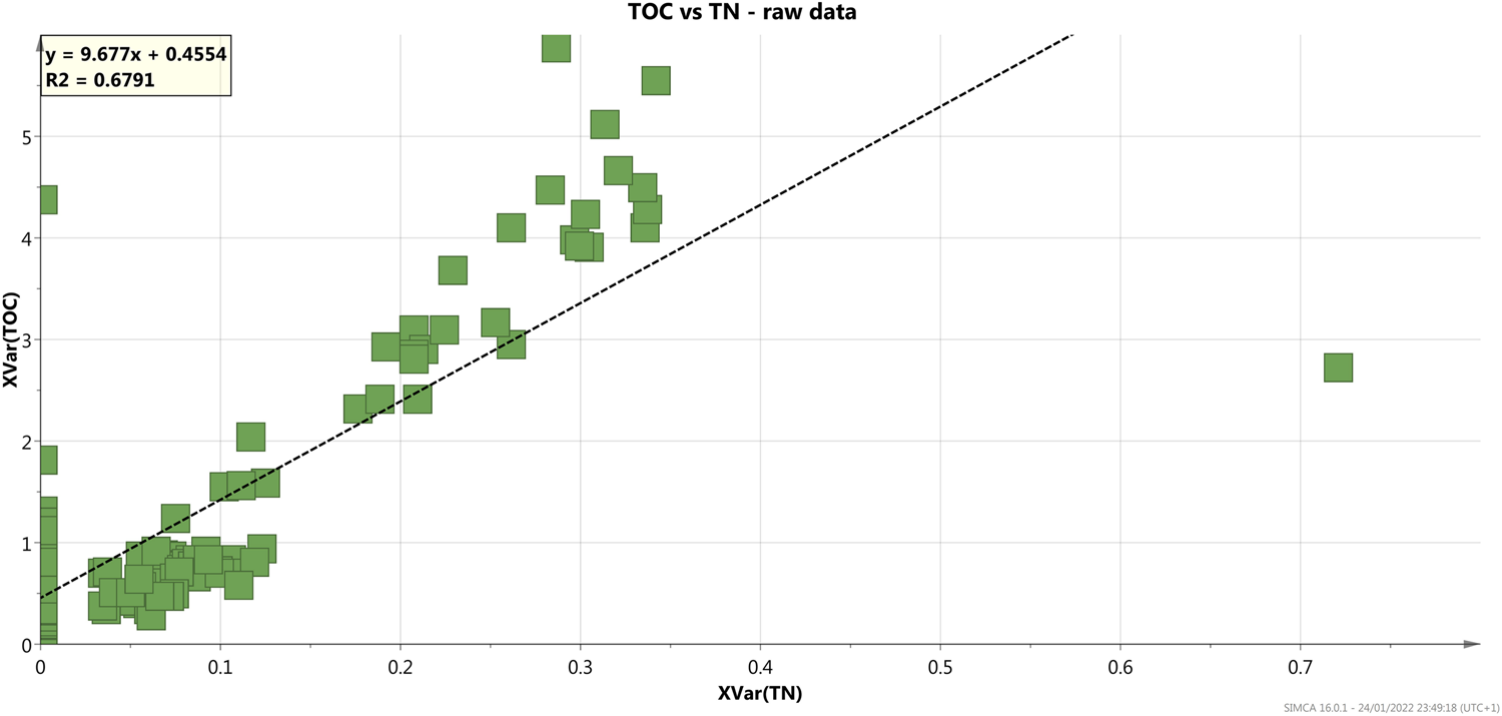
Correlation plots of TOC *vs* TN

The distribution of total nitrogen (TN) follows the TOC trend, as shown by the high correlation between TOC and TN plots (*R*^2^ = 0.679 at *p* = 0.05) (Fig. 3). The data show a positive correlation of the TOC concentration versus the finest grain size fractions (clay and/or silt). The close association of the two components can be explained by (1) the capacity of the finest particles to hinder the diffusion of the oxygen into the sediments, which induces the preservation of organic matter, and (2) the adsorption of organic particles onto the charged surfaces of the clay minerals (Mayer 1994; Hedges and Klein 1995; Cotecchia et al. 2021). In turn, the adsorption process helps to preserve the organic matter and gives rise to a generally positive correlation between TN or TOC and % finest sediment (Hedges and Klein, 1995).

#### Contaminant patterns

##### *Trace elements*

The concentrations of trace element are reported in Table S2 and in Figure S1. Cd and Hg resulted to be elements of high concern, both reaching maximum values in the middle offshore track (GN82 and GN77), with values of 8.38 mg/kg and 0.70 mg/kg, respectively. High concentrations of Cd were registered also near the CA coast (7.39 mg/kg in GN120) and in some samples located in the correspondence of the track of TA (8.07 mg/kg in GN34). Concentrations of concern were logged also for Pb that reached the highest value of 231.36 mg/kg near the coast of TA (at site GN36) as well as for other two elements, Cu and Cr, that logged the maximum amounts of 243.55 mg/kg and 749.73 mg/kg, respectively. High values were found also for Ni and Zn that reached the amounts of 73.62 mg/kg (GN82) and 386 mg/kg (at site GN36) respectively. It should be underlined that anomalous values were logged also for Fe, a ubiquitous lithogenic element, that reaches concentration of 10.3 W% (in GN4). The spatial distribution of some metals (such as Hg, Cd, Pb, Cr, Ni, and Cu) in general decreased gradually with distance from the coast, albeit some elements’ concentration remain high also in correspondence to the middle offshore area (MO) in the track between the two coasts, TA and CA. A similar spatial distribution was observed for the TOC (Table S2). The comparison of trace elements’ concentrations with sediment quality guidelines (SQGs) is reported in Table 1. Taking as a reference the chemical levels L1 (the lowest chemical level of reference) and L2 (the highest chemical level of reference) defined by the Italian Ministerial Decree 173/2016 for marine coastal areas, almost all elements (except Hg and Ni) exceed the national guidelines thresholds. Regarding international sediment quality guidelines (SQGs) ERM/ERL (effects range medium and effects range low), the concentration of only Cr, Pb, and Ni resulted higher than ERM values. Indeed, all elements (except Hg) exceeded PEL values indicated in TEL/PEL (threshold effects level and probable effects level) SQGs. According to Long et al. (1995a, b, c) concentrations below the ERL value represent a minimal effect range, i.e., a range below which adverse biological effect would rarely be observed, while the effects range median (ERM) value represent a potential range above which adverse effects on biological systems would frequently occur. Similarly the threshold effect level (TEL) and probable effect level (PEL), according to Mac Donald et al. (1996), are indicative values of probable toxic effects. The use of ERM/ERL and TEL/PEL is not considered as strict threshold for assessing the toxicity but a first attempt to link the bulk chemistry with toxicity according to literature (Long and Morgan 1990; Long et al. 1995a, b, 2000, 2006, EPA Method, 2001; SFEI 2003; Long and Sloane 2005; Birch 2018).

**Table 1:** National and international sediment quality guidelines for some trace elements. ^a^All concentrations are expressed in mg/kg dw; ^b^Min/max values verified in this study

SQGs	As^a^	Cd^a^	Cr^a^	Cu^a^	Hg^a^	Ni^a^	Pb^a^	Zn^a^
M.D.173/2016—L1	12	0.3	50	40	0.3	30	30	100
M.D.173/2016—L2	20	0.8	150	52	0.8	75	70	150
ERL (Long et al. 1995a, b, c)	8.2	1.2	81	34	0.15	20.09	46.7	150
ERM (Long et al. 1995a, b, c)	70	9.6	370	270	0.71	51.06	218	410
TEL (MacDonald et al. 1996)	7.24	0.68	52.3	18.7	0.13	15.9	30.2	124
PEL (MacDonald et al.^1996^)	41.6	4.21	160	108	0.7	42.8	112	271
This study^b^	**4.52–49.5**	**0.5–8.38**	**4.42–749**	**7.76–244**	**0.01–0.7**	**0.0–73.6**	**11.1–232**	**27.4–386**

##### Organic pollutants (PAHs, TPHs, OTs, PCBs)

The concentration levels of the selected PAHs are given in Table S2. Tukey box plot (Figure S2) reports minimum and maximum concentration values (ends of the whiskers), interquartile range (length of the box), and median (line through the box). The total concentration of Σ16PAHs range from 45.4 to 39,067 μg/kg. The highest PAH concentrations were logged within the area belonging to the CA coast (GN129 and GN131) and near TA coast where high concentrations of PAHs were registered in correspondence of the offshore track immediately after the TA shoreline (GN34 with values of 3517 μg/kg). As to the number of aromatic rings, the 4–6-ringed congeners resulted to be the most abundant in the whole area reflecting the predominance of a possible pyloric origin of the PAH contamination (Budzinski et al. 1997; Soclo et al. 2000; De Luca et al. 2004, 2005; Sprovieri et al. 2007; Mali et al. 2018). The highest concentration of 2–3-ringed PAHs was 2021 μg/kg, alongside CA coast. High concentration of 2–3-ringed PAHs (1060 μg/kg) has been registered also near TA coast. The 4–6-ringed PAHs resulted widespread in the whole area reaching values in the 18.2–3907 μg/kg range. The most abundant compounds resulted to be IDP, B[b]F, Phen, Fluo, Pyr (950 μg/kg, 924 μg/kg, 849 μg/kg, 786 μg/kg, and 561 μg/kg, respectively).

The PCB distribution was very variable (Table S2). From the 13 congeners analyzed, the highest variability was displayed by six congeners (PCB28, PCB 101, 128, 138, 153, and 180). The area of most concern in terms of PCB contamination was located near the Capri shoreline, where the highest PCB concentrations are registered. As to the VOCs and TPHs, the highest concentrations were 10.9 mg/kg and 686 mg/kg, respectively. The VOCs resulted also relevant (max 7.2 mg/kg) near Capri shoreline. It should be pointed out that relatively high level of VOCs (max concentration 7.5 μg/kg) was registered also in the middle part of the tract far away from the coasts. Regarding the OTs, as it can be noticed that from Table S2, they were quite equally distributed in the whole investigated coastal track albeit higher concentrations were registered in spot areas between Torre Annunziata and Capri.

The concentration of the mono butyltin congener (MBT) ranges from 3 to 16.71 μg/kg, whereas the concentration of the dibutyltin congener (DBT) ranges from 1.5 to 23.65 μg/kg, finally the concentration of tributyltin congener ranges from 0.75 to 13.87 μg/kg. Using butyltin degradation index (BDI), according to the formula reported in the Eq. (1), we can predict whether BT contamination is recent or no (Díez et al., 1997).

1 $$BDI=\frac{DBT+MBT}{TBT}$$

Values of BDI lower than 1 indicate that BT contamination is recent, and values of BDI higher than 1 indicate that there were no recent inputs of BTs to the sediments. The observed values of BTs in the present study (ranging from 1 to 156—Figure S4) indicate that the BT contamination in the study area is not recent except for some location near the TA coast (BT < 1), thus assuming that the BT contamination can be high enough to produce harmful effects on marine organisms (Antizar-Ladislao et al. 2011).

In general, as summarized in Table 2, PAHs and OTs never exceeded SQGs, contrary to PCBs that showed levels exceeding L2, ERM, and PEL values. Regarding TPHs, the maximum concentrations found was about ten times greater than L2 national SQGs.

**Table 2:** National and international sediment quality guidelines for some organic compounds. All concentrations are expressed in µg/kg dw

SQG	PAHs	TPHs	PCBs	OTs
173/2006—L1	900^a^		8^c^	5^e^
173/2006—L2	4000^a^	50,000	60^c^	72^f^
ERL (Long et al. 1995a, b, c)	4022^b^		23^d^	
ERM (Long et al. 1995a, b, c)	44792^b^		180^d^	
TEL (MacDonald 1996)	1684^b^		21.6^d^	
PEL (MacDonald 1996)	16770^b^		189^d^	
**This study**	**45.4–3963**^a^	**5–686**	**3.9–253**^**c**^	**4.5–68**^**f**^

^a^Ʃ16 US EPA PAHs

^b^Ʃ12 US EPA PAHs: in this sum BbF, BkF, IND, and B.g.h.i.P are not included

^c^Ʃ 13 PCBs considered in this study

^d^Total PCBs

^e^TBT

^f^Ʃ16 OTs

### Multivariate data analysis

#### Unsupervised PCA

A model with four principal components (PC1 56.2%, PC2 9.3%, PC3 7.3%, PC4 6%) explaining 77% of the total variance was obtained using the unsupervised PCA of the whole dataset to which was applied a log-transformation of the variables to normalize statistical distribution of the data (Eriksson et al., 2013). The goodness of the PCA model is verified by *R*^2^ and *Q*^2^ that in our case are *R*^2^ = 0.79 and *Q*^2^ = 0.71, indicative of good descriptive and predictive power of the model (values of (*R*^2^—*Q*^2^) < 0.3 confirm the adequacy of the PCA model (Eriksson et al. 2013)).

**Fig. 4:**
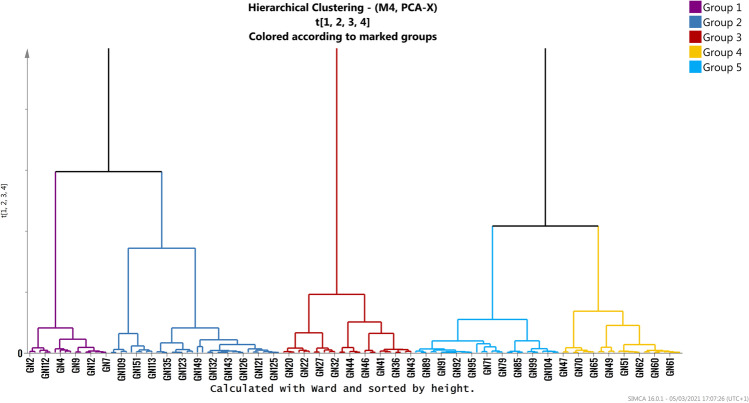
Dendrogram of hierarchical clustering of the PCA model

Hierarchical cluster analyses (HCA) was then performed on score matrix T according to Ward Method in order to identify and define cluster samples. The model distinguishes five cluster groups as shown in the dendrogram of Fig. 4. A first cluster (CL1 in violet) includes mainly samples collected near the shoreline of Torre Annunziata (TA) (sites from GN01 to GN14) and some sites of the other site of coastal track (GN112, GN120). The second cluster (CL2 in blue) includes samples collected on the other side of the investigated coastal track, starting from the shoreline of Capri going backward to the offshore area (mainly from GN104 to GN155). The third cluster (CL3 in red) includes samples collected in coastal track belonging to the area immediately after the shoreline of TA (sites from GN15 up to GN44). The fourth cluster (CL4 in yellow) includes samples collected at the last distal part of the TA track approaching the offshore area (from GN45 to GN70), while the last cluster (CL5 in light blue) includes samples located in the middle offshore track (MO) between TA and CA (sites GN71 up to GN105) (See Table S3 the member-list for each cluster).

**Fig. 5:**
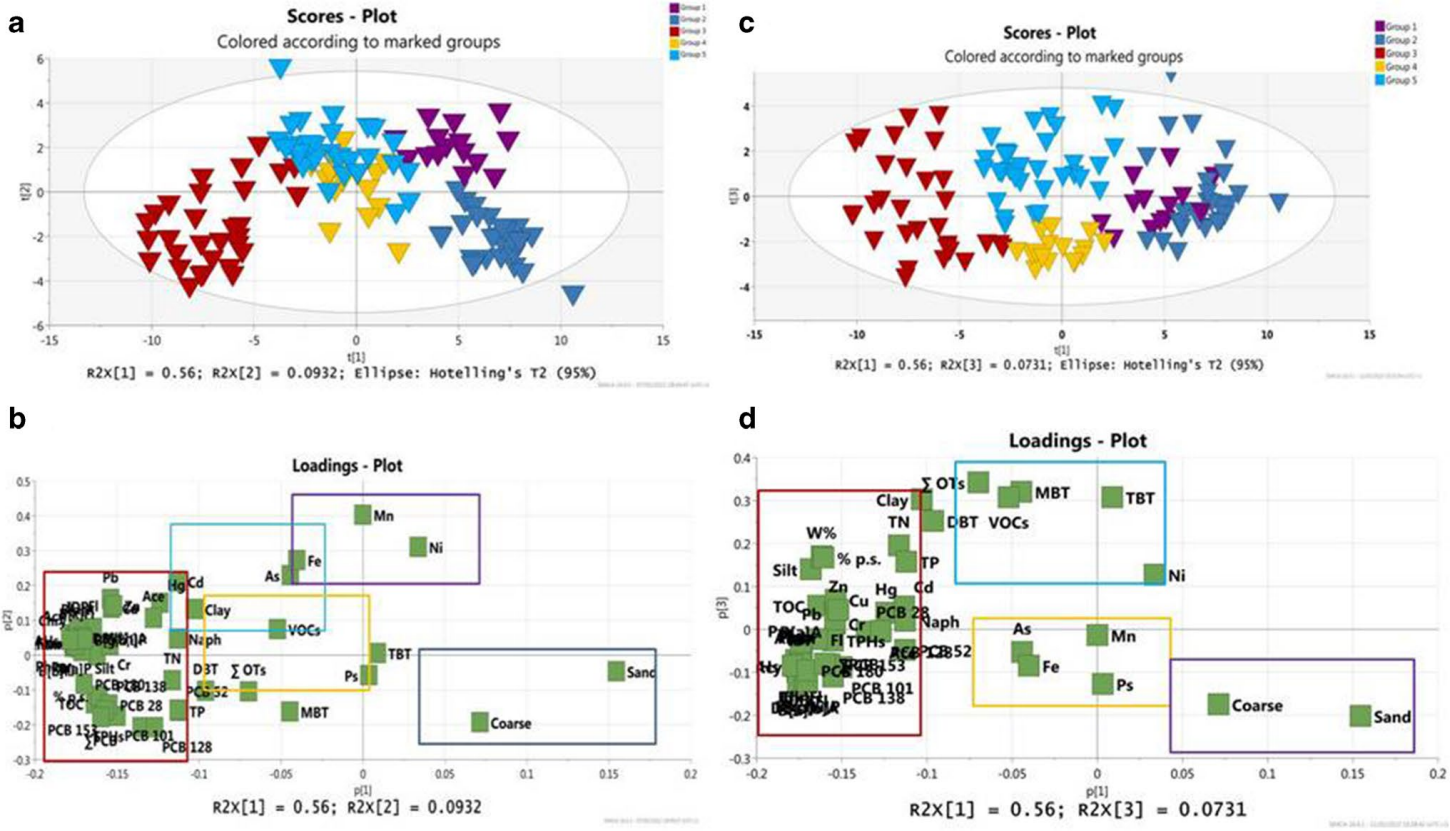
Projection of PC1 *vs* PC2 of score plot (**a**) and loading plots (**b**) of the selected model, colored according hierarchical clustering dendrogram. Projection of PC1 *vs* PC3 of score plot (**c**) and loading plots (**d**) of the model colored according hierarchical clustering dendrogram

By analyzing the projection of score and loading plots of the PCA model, colored according to the hierarchical clustering (Fig. 5a, b, c, d), it is possible to recognize that all mentioned clusters resulted well separated alongside the PC1 direction, which accounts for 56% of the total explained variance.

The first cluster (CL1 in violet) and second cluster (CL2 in deep blue) are both displayed on the PC1 *vs* PC2 hyperspace characterized by high positive values of PC1. The two clusters resulted separated from each other alongside the PC2: indeed, while the first cluster is displayed on the space characterized by the positive PC2 values, the second one is characterized by negative values of PC2. The third cluster (CL3 in red) falls on the PC1 *vs* PC2 space characterized by high negative values of PC1 and PC2, while the fourth (CL4 in yellow) and fifth cluster (CL5 in light blue) are located in the middle hyperspace of PC1 *vs* PC2, not so far from the origin. These two clusters (CL4 and CL5) are well separated along PC3 as it is shown in the projection plot of PC2 *vs* PC3 (Fig. 5c).

The variables governing this cluster distribution are revealed in the loading plots graphs (Fig. 5b, d) from which useful information can be deduced about the most discriminant parameters influencing different contamination pattern exposed. The loading plot p1 *vs* p2 reveals a correlation between the contamination pattern of CL1 and CL2 and the granulometry in particular with the coarser grain size fraction (sandy and coarse). These two clusters belong to shoreline coastal areas, CL1 to TA and CL2 to CA. The enrichment of shoreline sediments with coarser fractions (sandy and coarse) is supposed to be due to wave processes, which play an important role in determining superficial sediment distribution patterns, especially in the shallow water levels (a vertical range between 0.15 and 1.0 m), as documented by Malvarez et al. (2001). Besides the specific textural features, the samples of these two clusters display a different contamination pattern: the CL1 samples (TA shoreline) show positive correlation with Mn, As, Ni, Cd, and Fe, while samples of CL2 (CA shoreline) resulted mainly influenced by PCB contents and OT compounds (displaying both high negative loading values on PC2) (Fig. 5b)*.*

It seems that the contamination source varies in both areas probably due to different morphologies of the two coastal tracks, of Torre Annunziata and Capri, and due to different coastal land uses.

The third cluster (CL3) resulted of high concern being it associated with almost all contaminants, organic and inorganic such as petroleum hydrocarbons THPs, ∑PCB (including specific congeners, PCB180, PCB138, PCB153), almost all PAH congeners as well as toxic metals (Cu, Pb, Cr, Zn). The CL3 cluster is also distinguished by high values of TOC and nutrients (TN and TP) as well as high content of finest grain fraction (silt and clay). It is supposed that the area is interested by diffuse contamination thus needs deeper analysis.

The fourth cluster (CL4) includes a heterogeneous sample group counting mainly stations located on the distal part of the Torre Annunziata (TA). This cluster is characterized by a contamination pattern similar to the samples belonging to the fifth cluster (CL5) corresponding to the middle offshore (MO) area connecting TA area and CA area. The projection on PC1 vs PC2 plot reveals that samples of both clusters are highly associated with TN, Ni, and Mn, VOCs, and to a less extend by OTs congeners, all correlated to high content of finest grain fraction and W% (percentage of water content). Slight distinction among the contamination pattern of these clusters can be noticed on the projection of PC1 vs PC3 (Fig. 5c, d) that point out that CL5 cluster is associated mainly with VOCs, OTs (MBT, DBT and TPT), differently from the CL4 that results positively associated with As, Mn, Fe content and, to a less extent, with Ni.

#### Supervised PLS-DA

In order to get insights on the contamination sources within different clusters a supervised approach has been performed aiming to maximize the variance represented by each PC of the PCA model and to set up a descriptive model with high predictive capacity. The most widely used “supervised” approach is that based on partial least square regression (PLS) which, when the Y matrix is well defined in terms of class information (as in our case) it can be discriminated by the partial least squares (PLS-DA) (Brereton and Lloyd 2014). Subjecting dataset to the PLS_DA, a model with only 3 components was obtained that well discriminates the five previous random defined clusters (CL_s_) (Figure S5). The model showed *R*^2^ = 0.77 and *Q*^2^ = 0.67, indicative of good descriptive and predictive power of the model (generally, values of (*R*^2^—*Q*^2^) < 0.3 and *Q*^2^ > 0.5 are considered acceptable (Eriksson et al. 2013). The goodness of the PLS-DA model and its discrimination performance was validated also by means of misclassification table (Table 3), calculated by using the current work set as the prediction set. The misclassification table summarizes how the selected PLS model classifies the observations into the known classes. In our case, it confirms that 95.12% of the observations in the prediction set were correctly classified.

**Table 3:**
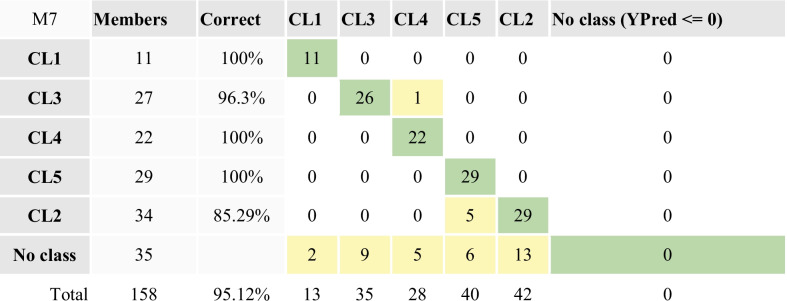
Misclassification table of the PLS_DA model

**Fig. 6:**
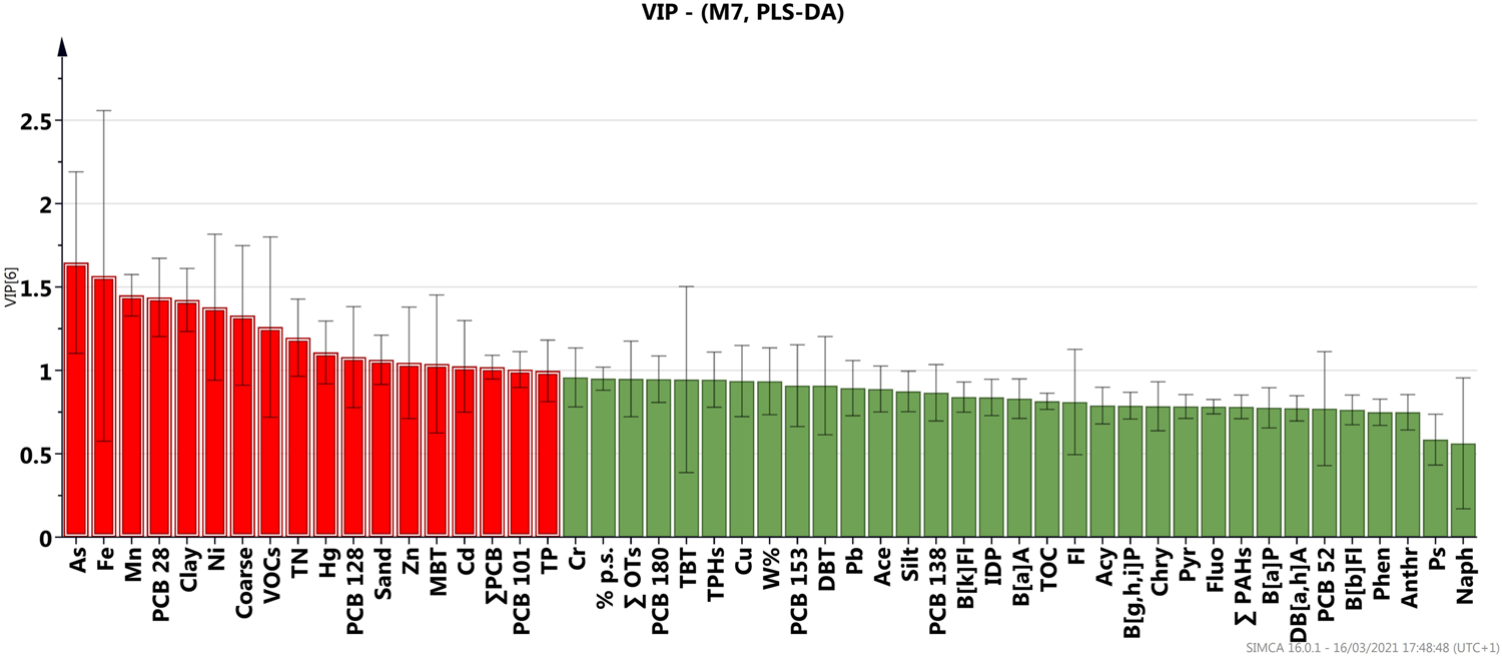
Variable importance projection in the PLS-DA model

The PLS_DA model points out different geographic areas supporting a better definition of sub-areas and their specific contamination pattern. A first geographic area is the marine track belonging to Torre Annunziata (TA) that includes the samples from the shoreline (GN1-GN15, included in the CL1 cluster), getting through the subsequent sites (GN20-GN44 included in CL3), up to the sites in the approaching the offshore area (GN45-GN70 belonging to CL4). This area (TA) resulted one of most concern in terms of contamination profile. A second geographic area (Capri, CA) is located on the other side of the investigated coastal track and belongs to Capri shoreline, including almost all sites of the cluster CL2 of PCA. The third area, middle offshore (MO), is located between (TA) and (CA), and it seems to be not interested by severe contamination loading. The variables that most control this clusters are reported in Figure 6 representing the values of the variable importance in the PLS projection, a statistical parameter indicating the influence of each variable on the model (variables with VIP > 1 are considered more relevant on the classification model).

**Fig. 7:**
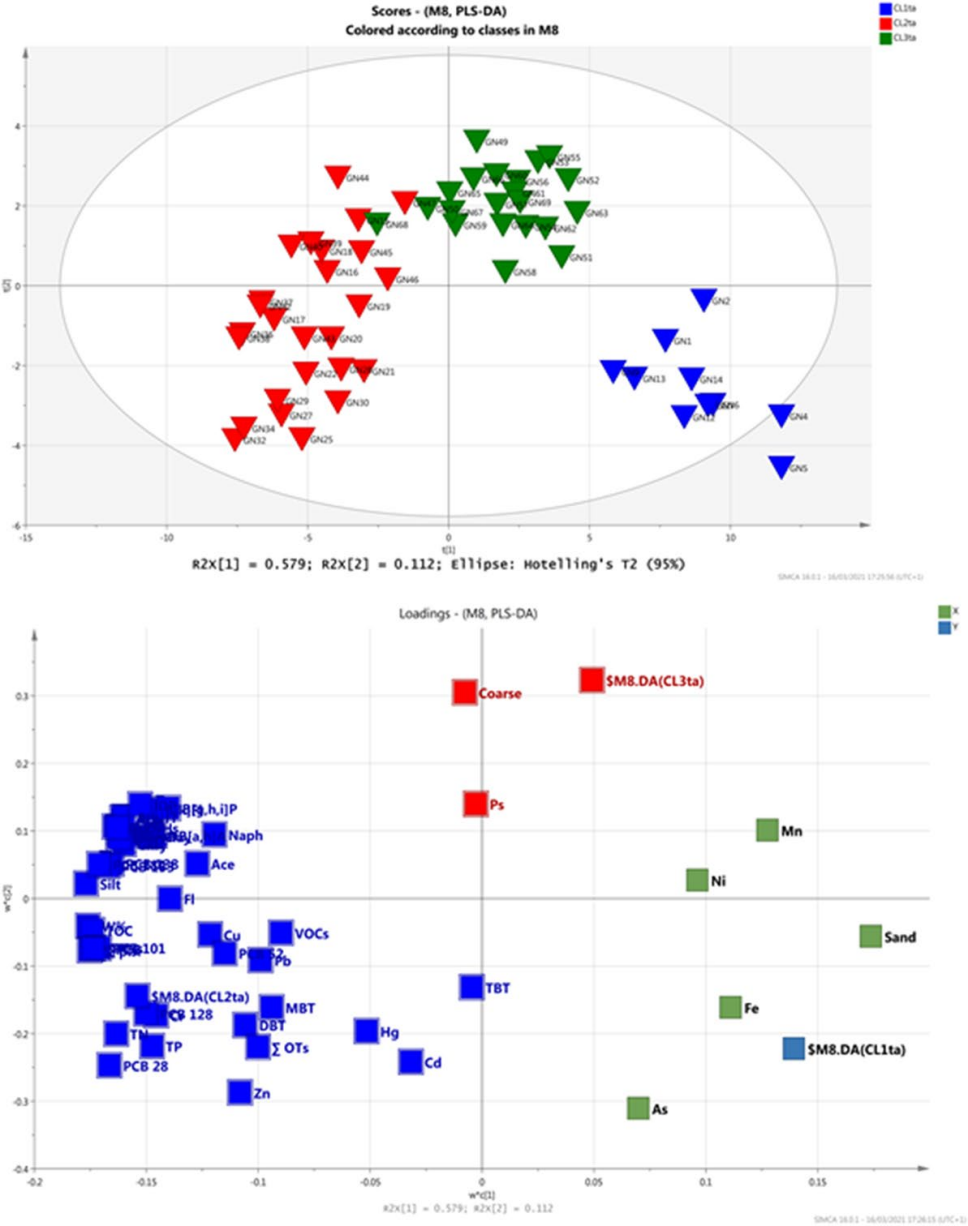
Scoreplot of t1/t2 of PLS-DA_TA_ model (**a**), loading plot of w1/w2 of PLS-DA_TA_ model (**b**)

Continuing more thorough investigations, aiming to understand the common factors (natural and or anthropogenic) governing each geographic area, supervised PLS_DA models using partial dataset of each defined sub-areas has been performed: first, a PLS_DA_TA_ model with three principal components was obtained using the partial dataset of Torre Annunziata (samples from GN1-GN70) (Fig. 7a, b). The model explains almost 77% of the total variance of the dataset and distinguishes three sub-cluster within TA, as shown in the 3D score plot and loading plot projections. The descriptive parameters (*R*^2^(cum) = 0.71) as well as the predictive power (*Q*^2^(cum) = 0.70) of the model confirmed the three random defined group classes as denoted by the misclassification table (Table 4) confirming that 100% of the observations in the prediction-set were correctly classified.

**Table 4:**
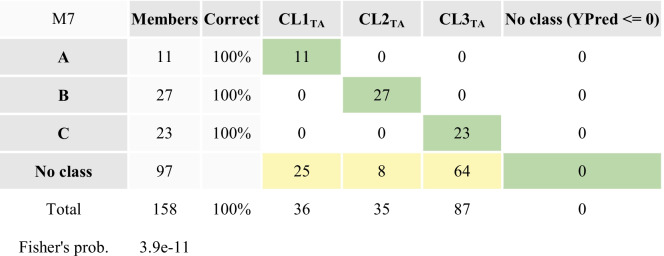
Misclassification table of the PLS_DA_TA_ model

The score and loading plots of the PLS_TA model (Fig. 7) provides useful information reflecting the peculiarities of three new sub-clusters (CL1_TA_, CL2_TA_, and CL3_TA_) belonging to TA track, which correspond to the original CL1, CL3, and CL4 cluster of the whole dataset, respectively.

The sub-cluster CL1_TA_, located near shore area, is characterized by sandy granulometry and by a prevalent inorganic contamination, mainly due to high metals concentrations (Fe, Mn, As, Ni). These contaminants are usually generated by tannery industries operating in the Sarno plain (De Pippo et al. 2006; Albanese et al. 2013). Nevertheless, natural processes related to the physical–chemical condition and hydrodynamic regime determined in the near shore area might also affect this contamination pattern, where pH and salinity are appropriate, iron and manganese oxides coagulate the fastest (Singh et al. 2009; Salazar et al. 2015). This occurrence could accelerate the deposition of some siderophilic and/or some chalcophilic metals as Ni and As, which resulted abundant in the sediment of this track of Torre Annunziata. On the other hand, under oxidizing conditions, metals in oxidizable fraction can also be re-released during the oxygenation of organic substances and/or sulfides and retained by Fe/Mn oxides. These hypotheses may explain the association of the elements As, Ni with Fe and Mn, constituents of ubiquitous oxy-hydroxides in sediment matrix. Finally, the concentration of As could have also volcanic origin (Albanese et al. 2013). Definitively multiple sources can be supposed for the cluster of these elements.

The sub-cluster CL2_TA_ includes stations GN15 to GN44 (countersigned in red in Fig. 7) and resulted positively associated with the silty sediments and high TOC content. This sub-cluster is highly associated with both organic and inorganic contaminants.

High concentration of specific congeners of PCBs and of high-ringed PAHs and relevant concentration of toxic metals precursors of organometallic complexes, such as Cd, Cu, Hg, Pb, and Cr, are also logged in this area.

The distribution of the contaminant is influenced by particle size and by organic matter (OM), due to organic matter degradation process affecting both pH and Eh values (Salomons and Förstner 1984; Mali et al. 2015, 2016, 2017a, b, c, 2018, 2019).

**Fig. 8:**
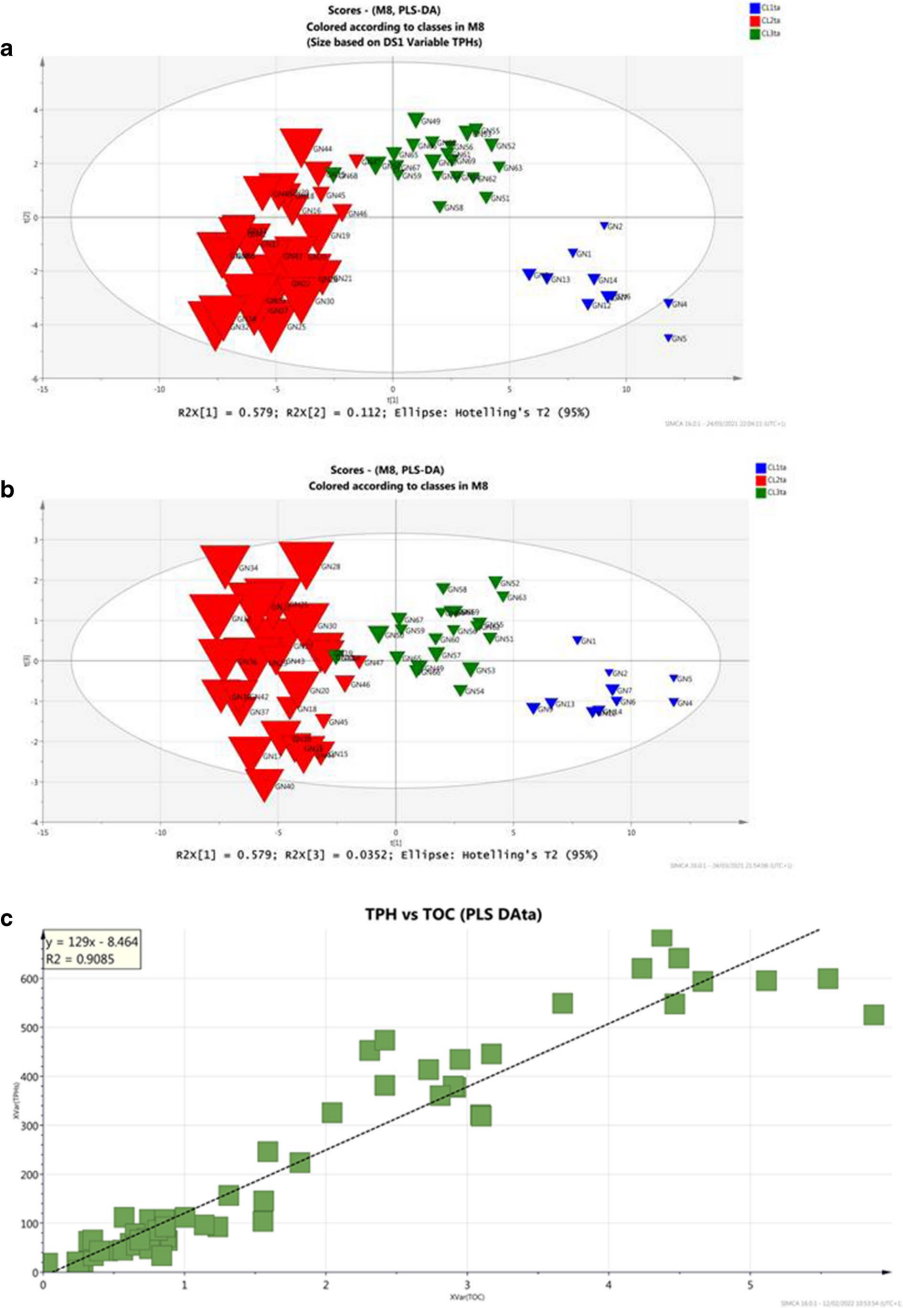
Distribution of TPHs (**a**), TOCs (**b**) in TA clusters and plot correlation of TOC *vs* TPHs (**c**). The symbol size in **a** and **b** graphs is proportional to TPHs and TOCs concentrations

The relationship of TOC values with the main contaminants shows significant positive correlation coefficients (at *p* < 0.05): TOC vs TPH, the Pearson’s correlation reaches *R*^2^ of 0.91 (Fig. 8c), TOC vs Pb and TOC vs Cu, both *R*^2^ > 0.50 and; c) less extent for TOC vs Cr. The circumstance led to suppose that contaminants’ trend is controlled by organic matter, probably due to exchange sorption, complexation, or chelation phenomena that increase thus their hazard (Baran et al. 2019). The contamination of this sub-cluster is supposed to be of anthropogenic origin and related to the human activities of Sarno River Plain. Indeed, the good correlation determined between Cr vs Ni and Cr vs Fe is supposed to be strictly correlated to Tannery district located in the Sarno River Plain (Albanese et al. 2013). The Sarno River discharges the contaminated water through its water flux into the marine basin of the Gulf of Naples. The hydrodynamic circulation influences the spatial and temporal distributions of contaminants in marine ecosystem especially through transport process of the finest sediment (Mali et al. 2017b; Malcangio et al. 2017). The transport of fine sediments from the river network towards the marine-receiving basin promotes not only the mere physical transport of the sediment and the associated contaminants, but also many chemical-geological-biological processes such as flocculation, precipitation, adsorption, redissolution, iron oxyhydroxides-driven process and other biogeochemical cycles (Rey et al. 2005; Vilas et al. 2010; Sollecito et al. 2018; Mali et al. 2015, 2018). Definitively, we can infer that the contamination sources of this sub-cluster are of anthropogenic nature, but they might be enhanced by the organic matter content and the depositional environment. The anthropogenic nature of the contamination in the area is supported also by the high concentration of specific PCB content, especially congeners PCB180, PCB 156, and PCB138 as well as of high-ringed PAHs (4–6 rings) that are caused by man-made combustion sources and activities (automobile emissions, incomplete combustion of fuel oils in heating systems, etc.).

Finally, the sub-cluster CL3_TA_, which includes stations from GN45 to GN70, results characterized by a coarse granulometry and reflects a decrease of the contamination patterns expected for sites approaching the offshore area. The persistence of some high concentration of VOCs in this area can be probably related to the capacity of such compounds to be associated with fine sediment that are more prone to be transported on long distances.

**Fig. 9:**
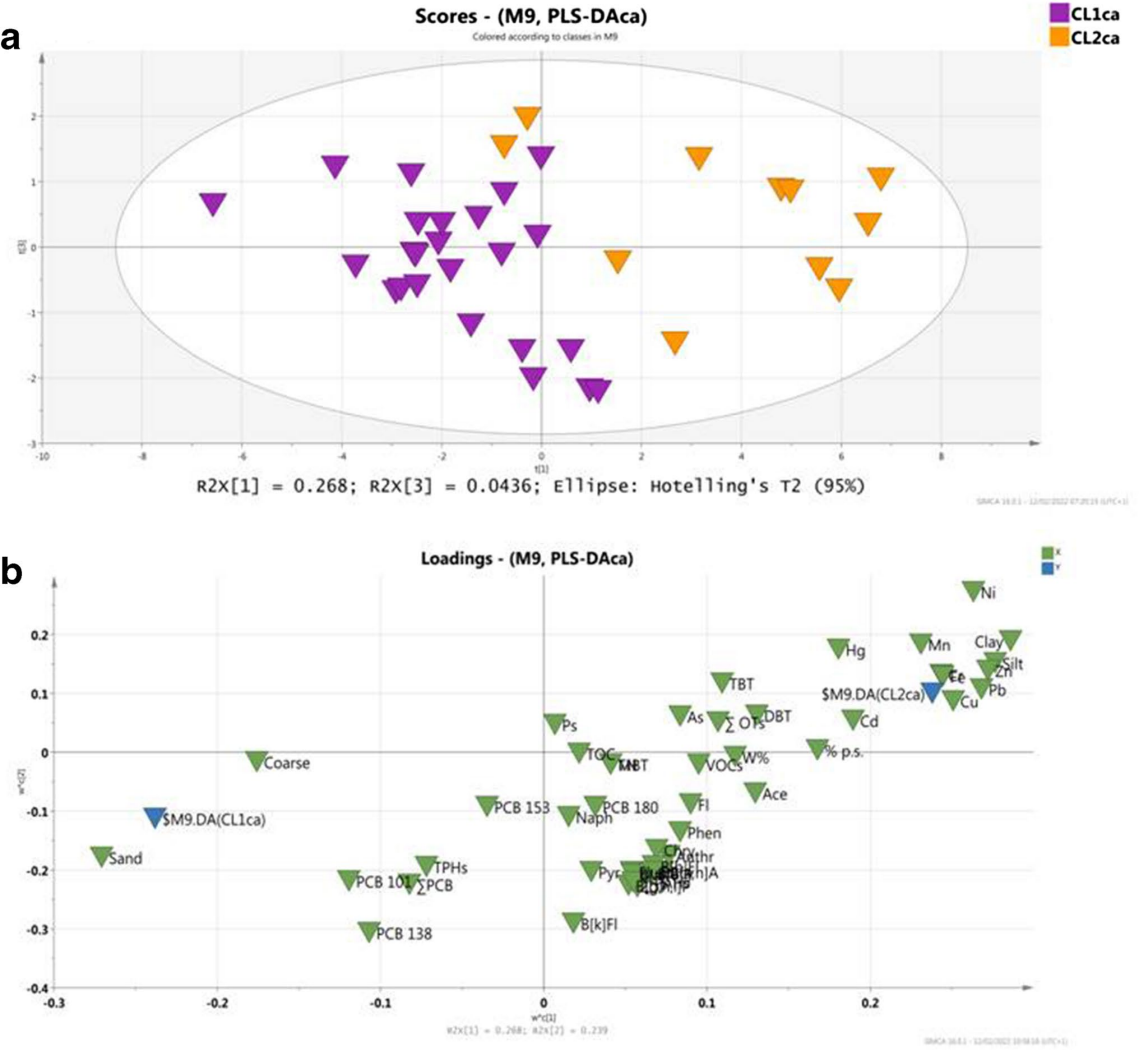
**a** Score plot of t1/t2 of PLS-DA_CA_ model, **b** loading plot of w1/w2 of PLS-DA_CA_ model

Capri data has also been analyzed with a PLS_DA_CA_ supervised technique, exploiting the partial dataset including samples from GN104 to GN155. The model PLS_DA_CA_ identified two new sub-clusters for the CA area (Fig. 9a, b): sub-cluster CL1_CA_ comprises stations from GN104 to GN120 and sub-cluster CL2_CA_, which includes the stations of Capri shoreline, from GN122 to GN157.

The sub-cluster CL1_CA_ displays a grain-size distribution controlled by silt and clay fraction and positively associated with Hg, VOCs, TBT, and to a less extent by Ni and Mn. The sub-cluster CL2_CA_ results characterized by organic contamination controlled mainly by total petroleum hydrocarbons (TPHs), some PCB congeners (PCB138, PCB101) and PAH congeners (Naph, BkFl) (Fig. 8c). The relevance of petroleum hydrocarbons, PCBs and PAH congeners, might be attributable to the fuel losses from traffic-vessels operating from/toward Capri Island. The contamination trend of the sub-cluster CL1CA samples, prevalence of VOC, OTs (mainly tributyltin), Ni and Mn, might be related the granulometry features of the sediments. The nature of the muddy sediments (finest > 63 μm) might exert a grain size effect. In addition, the ship traffic loading on the area and the effects on the current regime of the maritime route connecting Capri Island to the continent can probably cause the predominance of some OT compounds (known antifouling substances for protection of ships/vessels). Further investigations are needed to better understand the role of hydrodynamic conditions induced by ship traffic in the long-shore transport of the finest sediment and contaminants associated with this fraction.

## Conclusion

A complex dataset containing analytic data of 158 sampling sites in the Gulf of Naples between Capri Island and Torre Annunziata was subjected to multivariate techniques using a workflow analysis ranging from unsupervised techniques PCA and HCA to the supervised PLS-DA. This approach quickly enabled the discrimination of the main variables controlling the pollution pattern and permitted to identify and distinguish factors (natural and/or anthropogenic) affecting the spatial distribution along the study area. The adopted multivariate approach supported the identification of three different geographical areas in the investigated marine track and facilitate the recognition of the different factors governing the contaminant profiles. The area of Torre Annunziata (TA) and the area of Capri (CA) are both characterized by a severe pollution pattern of anthropogenic nature and the Middle Offshore area (MO) is less interested by contamination. The anthropogenic factors are related with the coastal land use, such as industrial activities (tannery industries) and intensive agricultural practices occurring alongside TA coast that influence the quality of the related sediments, while tourist ship traffic resulted to be the main anthropogenic factor loading on the CA coastline. As to the natural factors, the analyses highlighted the influence of the textural features in the distribution of inorganic contaminations, which resulted evident in the contamination profiles of both TA and CA shorelines. Interesting findings were discovered for the marine watershed that receives Sarno waters, in front of Torre Annunziata. Here, the effects of grain-size and organic matter content, coupled with the hydrodynamic regime generated by the Sarno River discharge, have strongly compromised the quality pattern not only on the near shore area but also on the whole maritime track in front of TA landing. The severe pollution profile found in this area can be reasonably explained by a contamination derived by the Sarno Plain that is transferred to the Gulf of Naples through Sarno River. The environmental parameters of the third identified area (MO) are controlled only by the natural factors related mainly to the current circulation. Further investigations are warranted to understand the induced circulation from the ship traffic on the Capri Island side to Campania Landing.

In conclusion, the present multivariate statistical approach brings advantages over the punctual numerical inspections, especially in case of complex datasets, and helps researchers understand the main trends in the variability of quality indicators, that can be selected as “summary indices” to assess the contamination status of the study area and to orient deeper investigations and further prevention actions.

## Supplementary Information

Below is the link to the electronic supplementary material.

Supplementary file1 (DOC 108 KB)

Supplementary file2 (DOC 197 KB)

Supplementary file3 (DOC 228 KB)

Supplementary file4 (DOC 1451 KB)

Supplementary file5 (PDF 6235 KB)

Supplementary file6 (DOC 3404 KB)

Supplementary file7 (XLS 39 KB)

Supplementary file8 (XLS 182 KB)

Supplementary file9 (XLS 33 KB)

## Data Availability

The datasets used and analyzed during the current study are available from the corresponding author on reasonable request.
