# Supplementary material for: Multivariate tools to investigate the spatial contaminant distribution in a highly anthropized area (Gulf of Naples, Italy)
Source: Environ Sci Pollut Res Int. 2022 Apr 9;29(41):62281–98. doi: 10.1007/s11356-022-19989-z (PMC9464125; doi:10.1007/s11356-022-19989-z)
Supplement: Supplementary file 1 — Supplementary file1 (DOC 108 KB) [file 11356_2022_19989_MOESM1_ESM.doc]

All analyses were performed under analytical quality control procedures that included the processes of certified or in-house reference materials and a blank sample with every batch of samples.

**PAHs and PCBs analysis:** 2 grams of freeze-dried samples were extracted with n-hexane/acetone (1:1 v/v) using a microwave digestion system (MARSX CEM Corporation, Matthews, NC) according to EPA Method 3546. The collected extracts, filtered through PTFE filters of 5µm pore size (Millipore, Bedford, USA), were split into two fractions and concentrated to a small volume by Turbo Vap ®II (Biotage, Uppsala, Sweden). Clean-up for PAHs and PCBs, were carried out on the Silica Gel column (EPA Method 3630C) and Florisil cartridge (EPA Method 3620C), respectively. More information on these steps were reported in Cardellicchio et a. 2007 and Di Leo et al. 2016. Purified samples were analyzed with an Agilent 7890A gas chromatograph equipped with 7693 autosampler, and coupled to an Agilent 5975C mass spectrometry) (GC-MS Agilent Technologies, inc. Santa Clara, CA, USA) following to USEPA method 8270E. 5 μL of analyte was injected into a PTV injector in solvent vent mode. All analytes were separated on a Select PAH column (30m x 0.25 mm i.d. x 0.15 µm film thickness, Agilent Technologies). The mass spectrometer was used in electronic impact mode (70 eV electron energy) and ion source, quadrupole, and transfer line temperatures were set at 230, 150, and 300 °C, respectively. Quantifications were performed in selected ion monitoring (SIM) mode using three ions for each PAH compound. PAHs calibration mix, deuterated internal standards (naphthalene-d8, acenaphthene-d10, phenanthrene-d10, chrysene-d12, and perylene-d12), and surrogate standards (anthracene-d10 and benzo[a]anthracene-d12) were purchased from Merck© as well as all chromatographic pesticide grade solvents and certified reference material (Merck s.p.a., Milan, Italy). The recovery, determined by analyzing standard reference materials NIST® SRM® 1941b, was 69-115% and 72-95% for PAHs and PCBs, respectively. PAHs relative standard deviation was included between 5%-11% (for triplicate analyses) while method detection limits calculated based on a signal-to-noise ratio of 3:1, ranged from 0.5 to 1.2 µg/kg d.w. As for PCBs, method precision was less than 8% while the method detection limit was 0.3 μg/kg dw.

**OTs analysis:** freeze-dried sediments were analyzed following the analytical method reported in ICRAM Method, 2001according to Morabito et al. (1995). Briefly, 1 g of freeze-dried sample was added with tropolone in methanol and concentrated hydrochloric acid. The supernatant was placed in a separating funnel and extracted with dichloromethane. The organic extract was then evaporated almost to a small volume in iso-octane and reacted with pentyl magnesium bromide (Grignard reagent). The derivatives, cleaned up on the activated silica gel column, were finally analyzed, in SIM mode, by the GC-MS apparatus described above. Monobutyltin, dibutyltin), tributyltin, Tripropyltin, as internal standard, tetrabutyltin as surrogate, pentylmagnesium bromide in 2 M in diethyl ether (Grignard reagent), silica gel, and all the other chemicals were obtained from Merck© (Milan, Italy).The accuracy of the analysis was verified by processing standard reference materials BCR 462 and in-housespiked sediment. Recovery was between 79 e 92% while the method detection limit ranged from 1.5 to 6.0μg/kg dw.

**TPHs analysis:** the concentration of TPHs was determined as a sum parameter of resolved and unresolved components eluted from the GC capillary column between the retention times of n-decane and n-tetracontane according to UNI EN ISO 16703:2011 Method and ISPRA Method 75/2011. Briefly, 10 g of the air-dried sediment samples were extracted with n-hexane/acetone (1:1 v/v) solution in a microwave oven (EPA Method 3546). After extraction samples were filtered through a GF/F filter, reduced to a small volume, and added with 10 ml of heptane. Clean up was carried out on Florisil SPE cartridge with 2 cm of Na_2_SO_4_ on the top and, the eluate determined in GC/FID (Agilent 7890B gas chromatograph equipped with 7693 autosampler) in splitless mode using a DB-1HT+DG capillary column (15 m × 0.32 mm,i.d..x 0.10 µm film thickness,Agilent Technologies) and ultrapure helium (2ml/min) as carrier gas. The accuracy of the analysis was verified by processing certified reference material ERM-CC015a. TPHs were expressed in mg/kg dw.

**VOC analysis:**sedimentswere preparedaccording to EPA Method 5021A using a headspace method and analyzed by GC/MS (EPA method 8260). Briefly, about 5 g of wet sample were added to a 20 mL headspace vial containing 10 ml of matrix modifier (saturated solution of NaCl with 0.2% of phosphoric acid). The vial was heated to85°C for 20 min and after vial equilibration time (35 min), a portion of the headspace above the sample was introduced into a GC-MS system (Electron TRACE GC with Electron DSQ, Thermo Scientific™) equipped with Thermo Scientific TriPlus 300 Headspace autosampler. The injection was in split mode (split ratio 10:1) and chromatographic separation was carried out on a DB-624 column (60 m × 0.25 mm i.d. x 1.4 μmfilm thickness, Agilent Technologies) with a Helium flow of 1.2 ml/min. The mass spectrometer was used in electronic impact mode (70 eV electron energy) and ion source, quadrupole, and transfer line temperatures were set at 230/300°C, 150°C, and 200 °C, respectively.The MS data were acquired in SIM according to EPA method 8260.The internal calibration standards (fluorobenzene, chlorobenzene-d5, and 1,2-dichlorobenzene-d4), surrogate standards (toluene-d8, 4-bromofluorobenzene), and VOCs stock standard solutions (VOCs listed in EPA Method 5021A)were purchased from o2si smart solution (North Charleston, SC). The RSD% of VOCs standard injection was in the range of 2.5 to 4.9 % for triplicate analysis. The method recovery, evaluated by spiking appropriate amounts of calibration standards into sediment samples, previously analyzed, was between 69 and 124%. Results were expressed in dw, which was calculated from the percentage weight loss obtained after drying at 105° C the wet sediments.

**Metal and metalloids:** ForHg, Cd, Pb, As, Cr, Cu, Ni, Zn, Fe, Mn determination, acid digestion was carried out with microwave digestion system MARSX CEM. According to EPA Method 3052, nitric acid and hydrofluoric acid were added to 0.25 g of freeze-dried sample. After digestion and cooling below the extractor hood, samples were treated with boric acid, filtered, diluted to 50 ml with ultrapure water, and analyzed by inductively coupled plasma mass spectrometry (ICP-MS model Elan 6100 DRC Plus, Perkin Elmer, Norwalk, CT, USA) following EPA Method 6020. All the chemicals used in sample treatments were of ultra-pure grade (Merck s.p.a., Milan, Italy), and all the glassware was cleaned prior to use with 10 % v/v HNO_3_ and rinsed with Milli-Q® water. For triplicate analysis, the relative standard deviations ranged from 5 to 10 %. The accuracy and precision of the analytical procedures have been checked by analyzing a certified reference marine sediment IAEA-356 (Buccolieri et al. 2006). Analytical results indicate a good agreement between the certified and found values with metals recovery between 88-100%.

References

Buccolieri A, Buccolieri G, Cardellicchio N, Dell’Atti A, Di Leo A, Maci A (2006) Heavy metals in marine sediments of Taranto Gulf (Ionian Sea, Southern Italy). Mar Chem 99:227-235.

Cardellicchio N, Buccolieri A, Giandomenico S, Lopez L, Pizzulli F, Spada L (2007) Organic pollutants (PAHs, PCBs) in sediments from the Mar Piccolo in Taranto (Ionian Sea, Southern Italy). Mar Pollut Bull 55:451-458

Di Leo, Annicchiarico C, Cardellicchio C, Cibic T, Comici C, Giandomenico S, Spada L (2016)Mobilization of trace metals and PCBs from contaminated marine sediments of the Mar Piccolo in Taranto during simulated resuspension experiment. Environ Sci Pollut Re 23:12777-12790.
