# Supplementary figures and images for: Multivariate tools to investigate the spatial contaminant distribution in a highly anthropized area (Gulf of Naples, Italy)
Source: Environ Sci Pollut Res Int. 2022 Apr 9;29(41):62281–98. doi: 10.1007/s11356-022-19989-z (PMC9464125; doi:10.1007/s11356-022-19989-z)

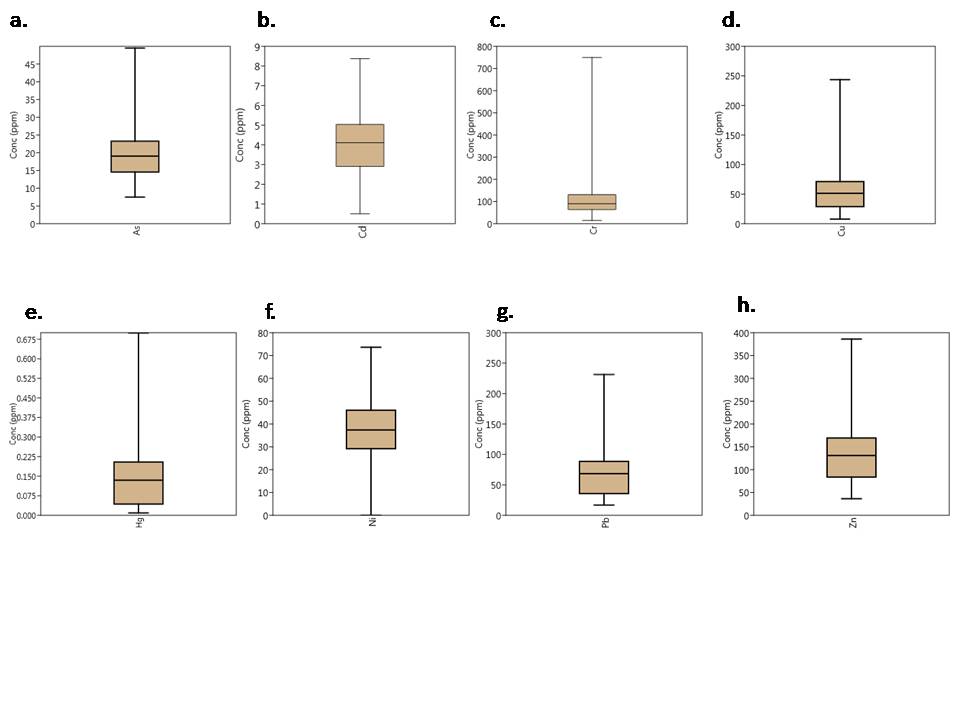


**Figure S1.** Box plot charts of total trace element concentration in the study area

Supplement: Supplementary file 2 — Supplementary file2 (DOC 197 KB) [file 11356_2022_19989_MOESM2_ESM.doc]

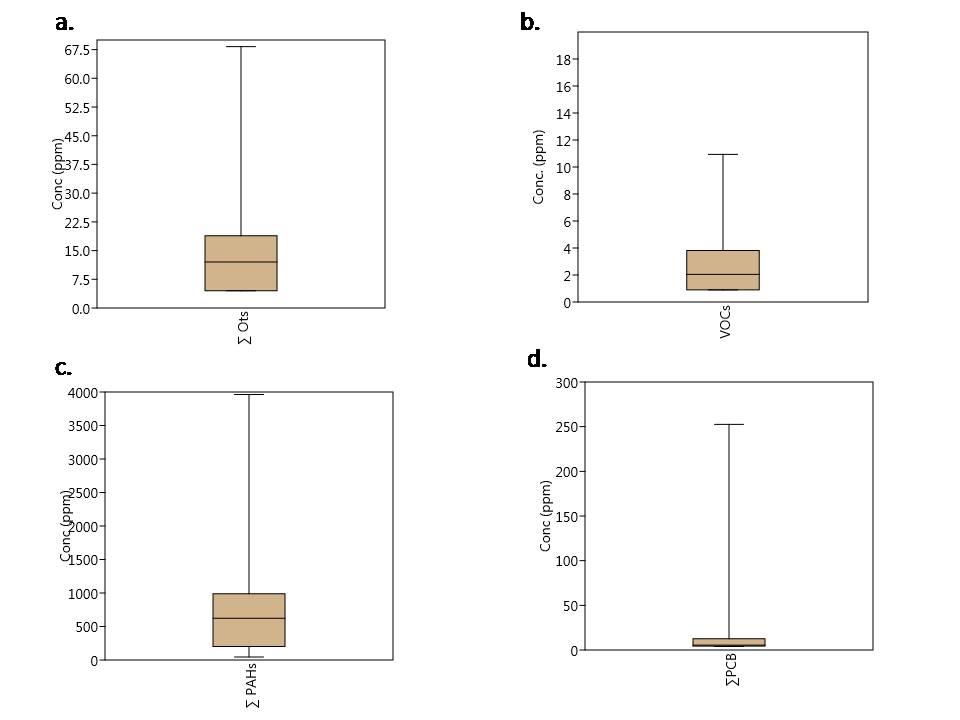


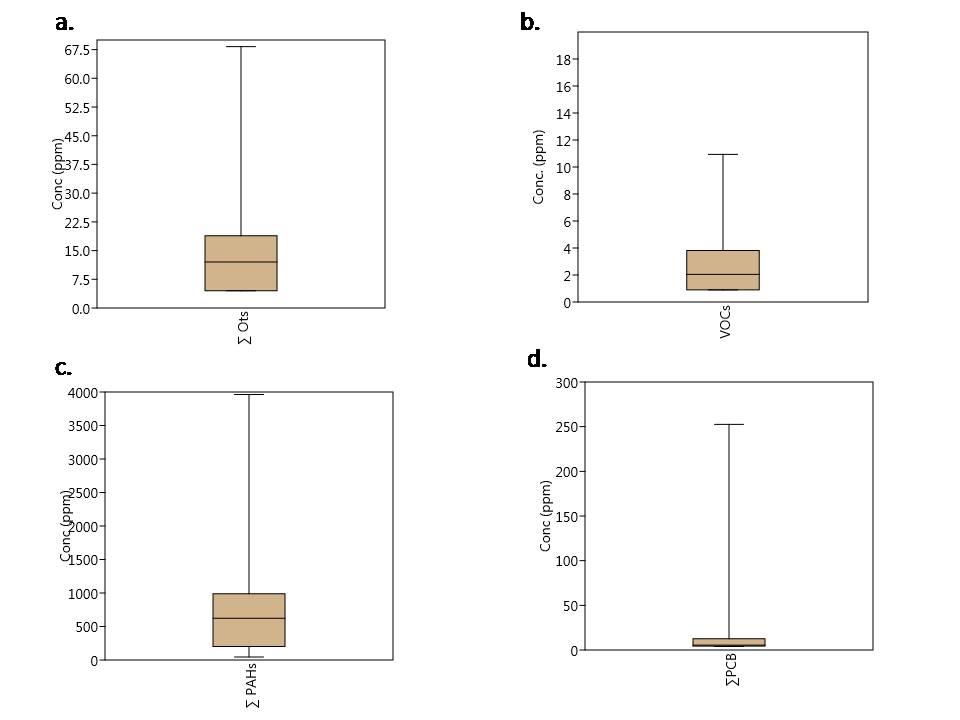


**Figure S2**. Box plot chart of ∑OTs, ∑VOCs, ∑PAHs and ∑PCBs

Supplement: Supplementary file 3 — Supplementary file3 (DOC 228 KB) [file 11356_2022_19989_MOESM3_ESM.doc]

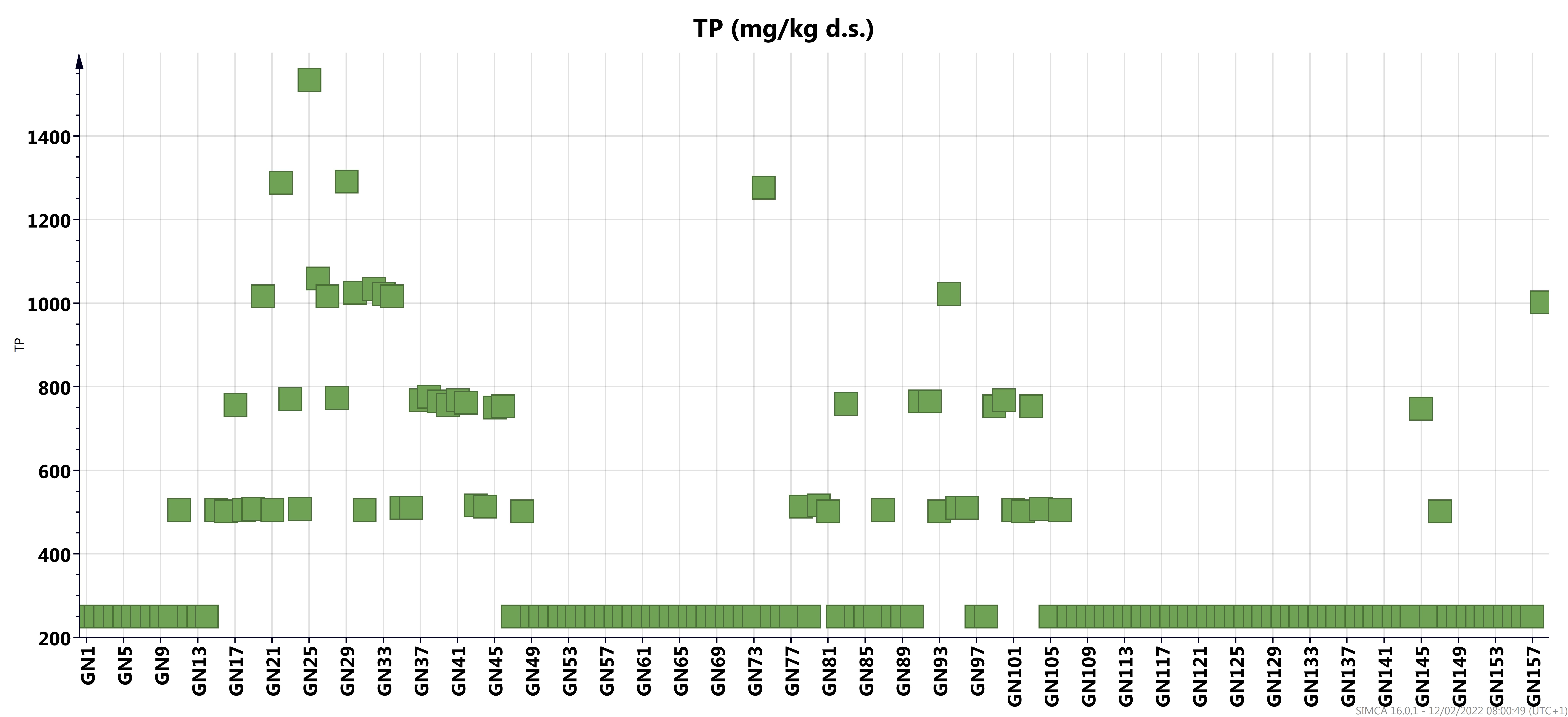


**Fig. S3**. Distribution of total phosporous in the study area

Supplement: Supplementary file 4 — Supplementary file4 (DOC 1451 KB) [file 11356_2022_19989_MOESM4_ESM.doc]

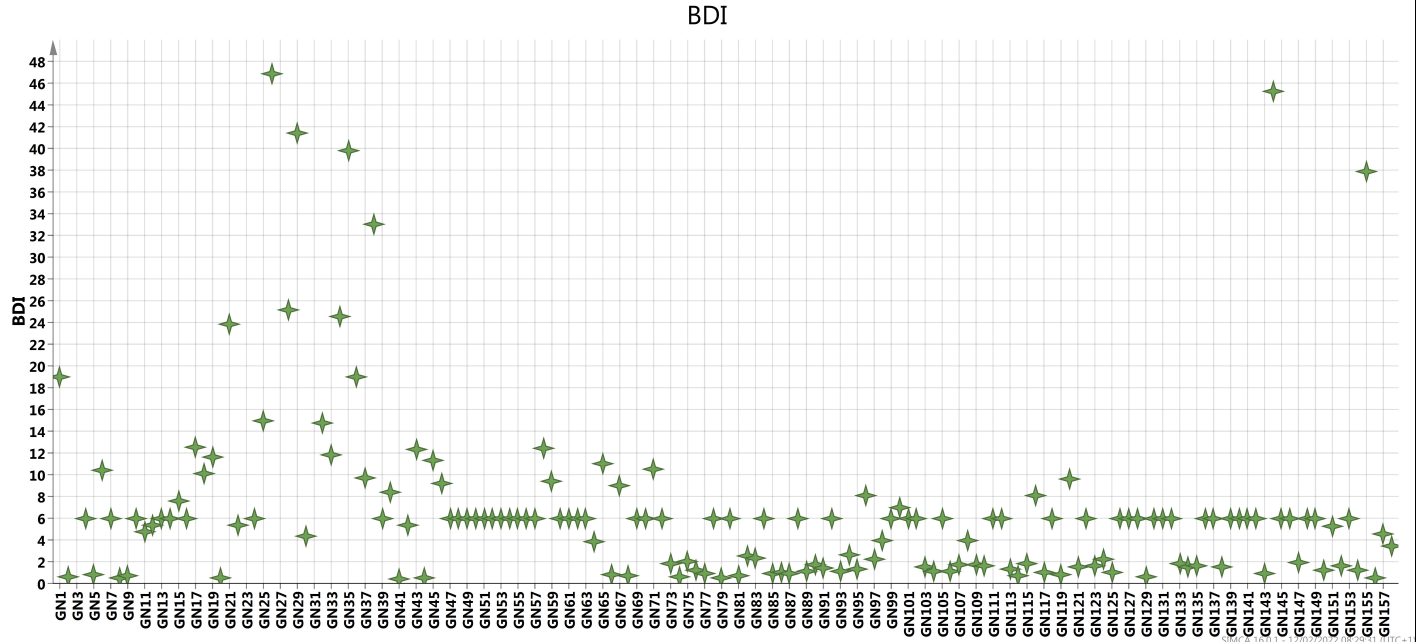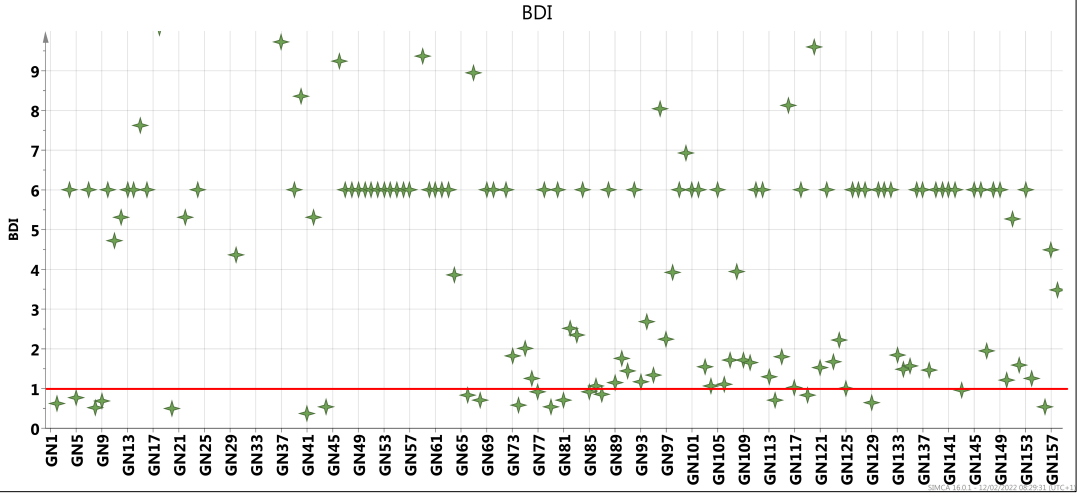

Supplement: Supplementary file 5 — Supplementary file5 (PDF 6235 KB) [file 11356_2022_19989_MOESM5_ESM.pdf]
