# Supplementary material for: Multivariate tools to investigate the spatial contaminant distribution in a highly anthropized area (Gulf of Naples, Italy)
Source: Environ Sci Pollut Res Int. 2022 Apr 9;29(41):62281–98. doi: 10.1007/s11356-022-19989-z (PMC9464125; doi:10.1007/s11356-022-19989-z)
Supplement: Supplementary file 6 — Supplementary file6 (DOC 3404 KB) [file 11356_2022_19989_MOESM6_ESM.doc]

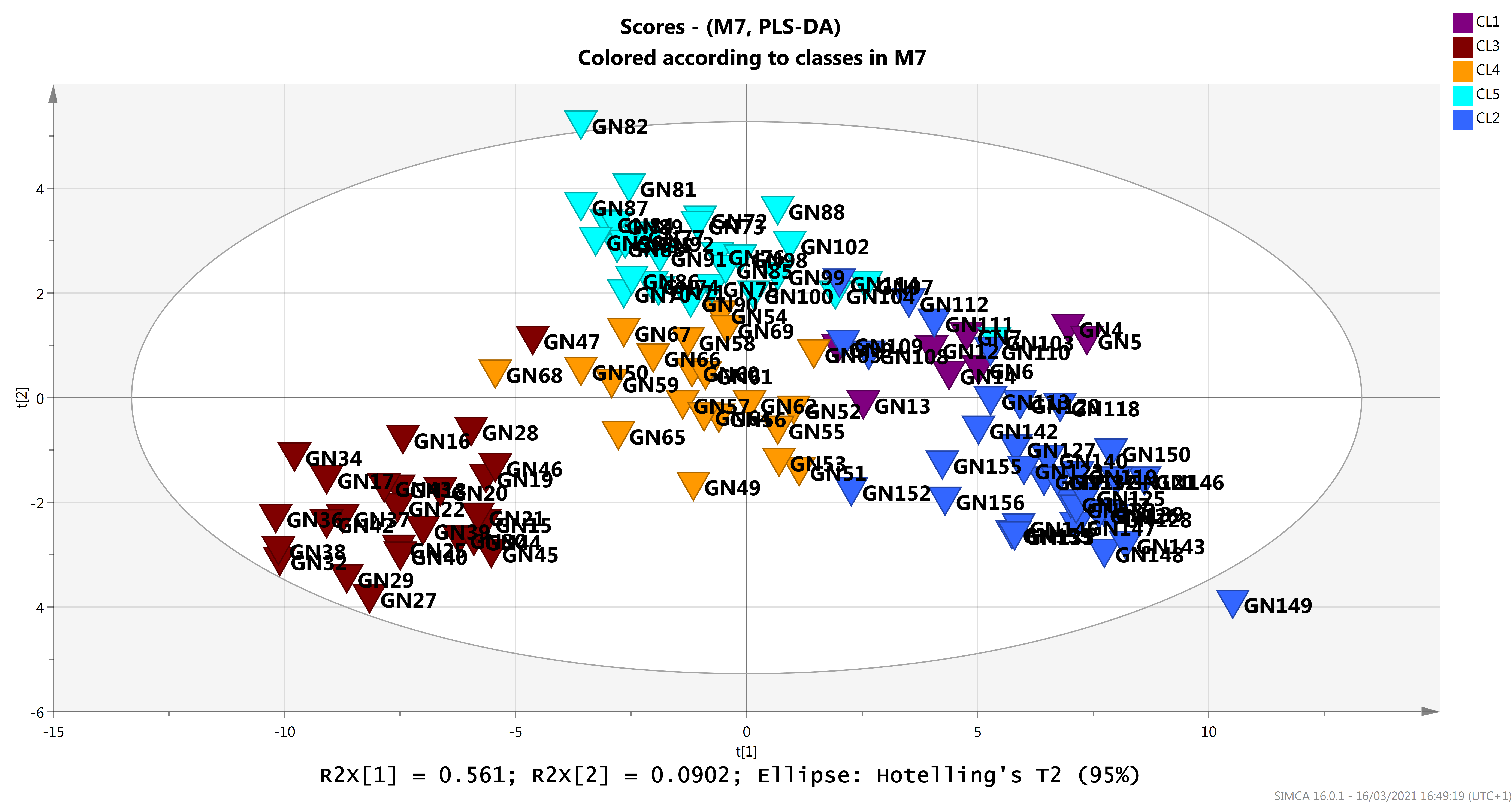

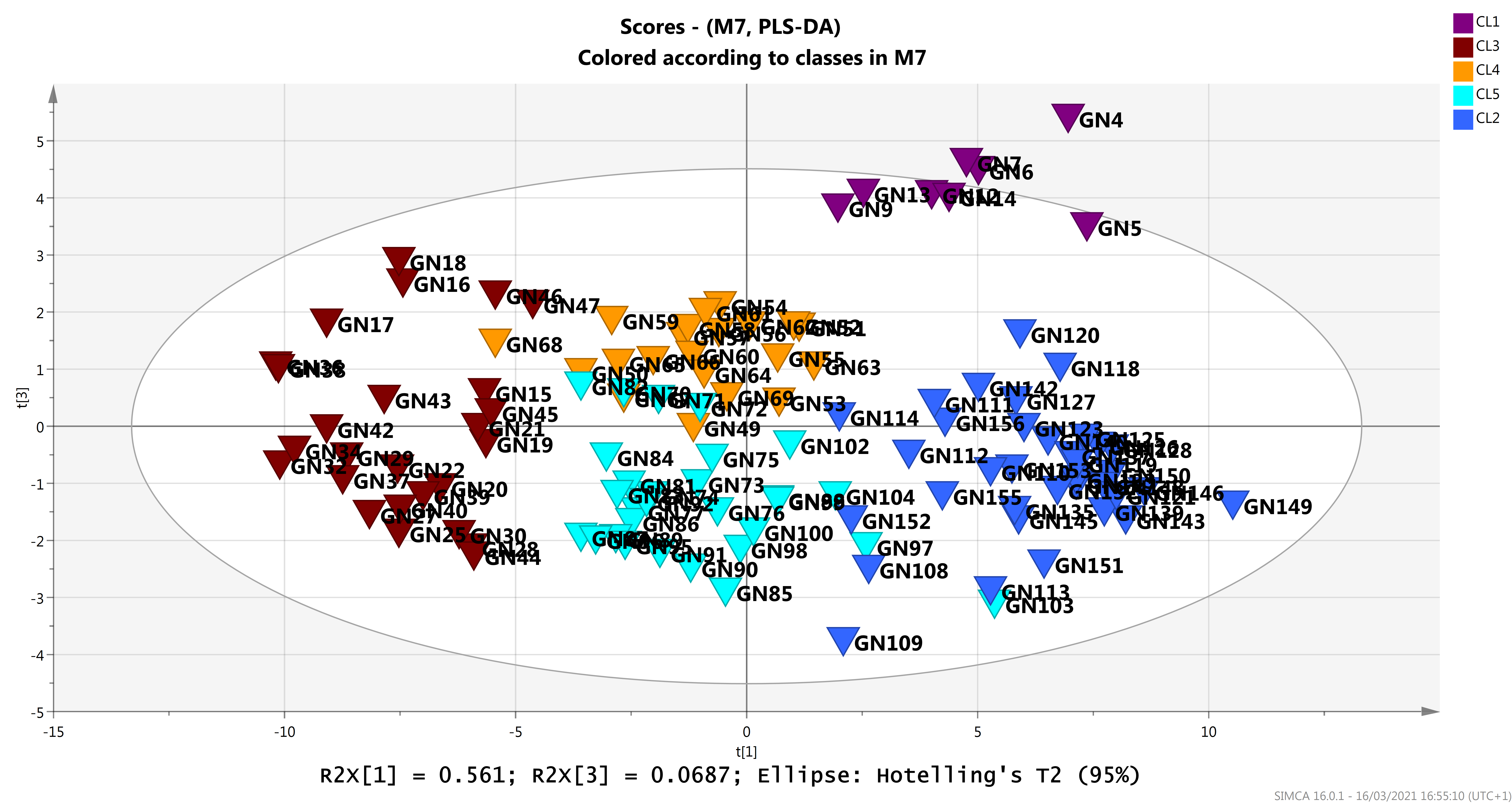


c. d.


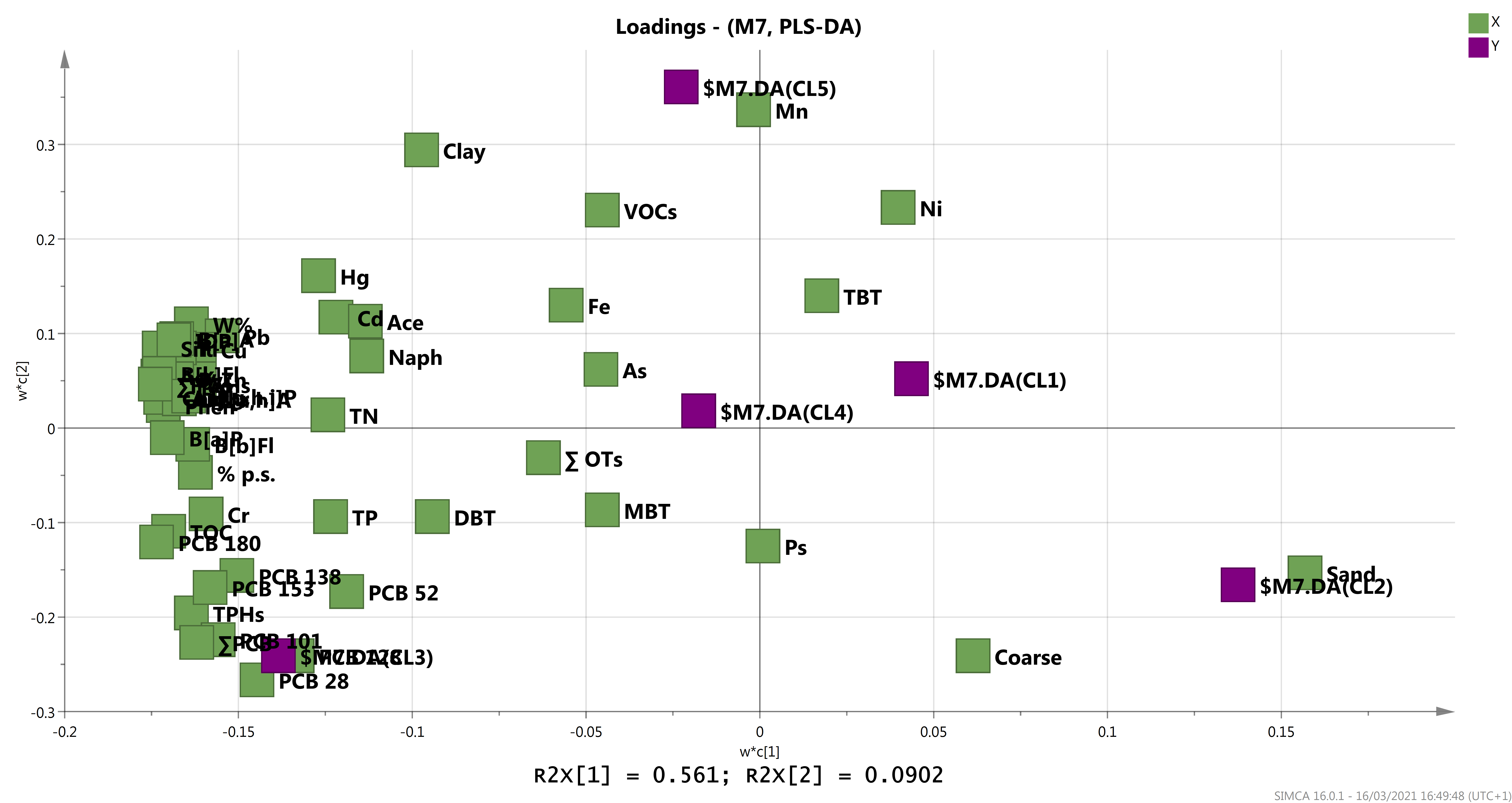

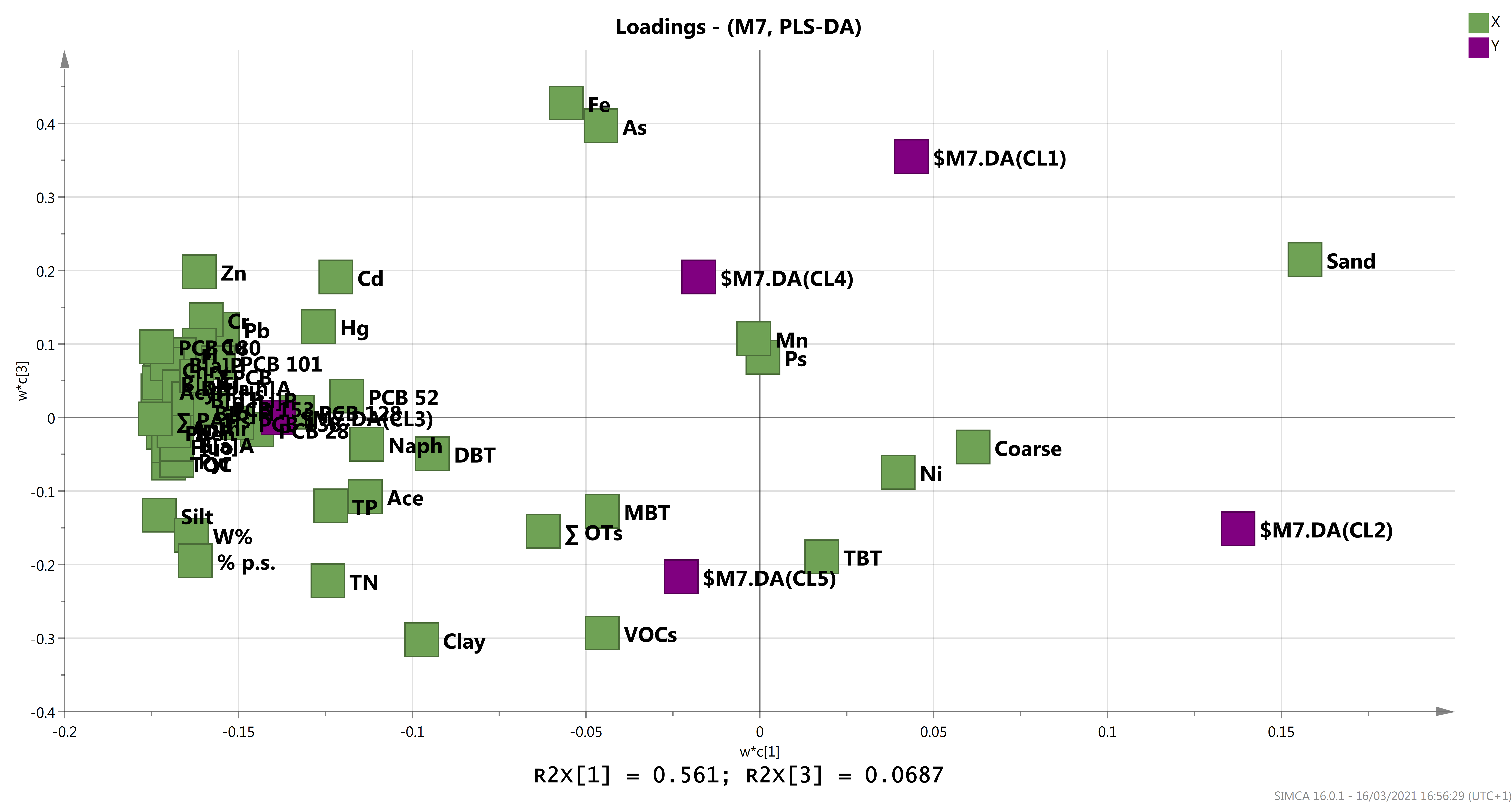


**Figure S5.** Score plot projection of PLS_DA model for PC1 vs PC2 (a) and for PC1 vs PC3 (b). Loading Plot projection of PLS_DA Model for PC1 vs PC2 (c) and for PC1 vs PC3 (d).
